# PARP-1 Regulates Metastatic Melanoma through Modulation of Vimentin-induced Malignant Transformation

**DOI:** 10.1371/journal.pgen.1003531

**Published:** 2013-06-13

**Authors:** María Isabel Rodríguez, Andreína Peralta-Leal, Francisco O'Valle, José Manuel Rodriguez-Vargas, Ariannys Gonzalez-Flores, Jara Majuelos-Melguizo, Laura López, Santiago Serrano, Antonio García de Herreros, Juan Carlos Rodríguez-Manzaneque, Rubén Fernández, Raimundo G. del Moral, José Mariano de Almodóvar, F. Javier Oliver

**Affiliations:** 1 Instituto de Parasitología y Biomedicina López Neyra, CSIC, Granada, Spain; 2 IBIMER, Centro de Investigaciones Biomédicas, Universidad de Granada, Granada, Spain; 3 Departamento de Bioquímica y Biología Molecular I, Universidad de Granada, Granada, Spain; 4 Departamento de Anatomía Patológica, Universidad de Granada, Granada, Spain; 5 Institut Municipal d'Investigació Mèdica (IMIM), Universitat Pompeu Fabra, Barcelona, Spain; 6 Pfizer-University of Granada-Junta de Andalucía Centre for Genomics and Oncological Research (GENYO), Granada, Spain; 7 Unidad de Anatomía Patológica, Complejo Hospitalario y Áreas Sur y Noreste de Granada, Granada, Spain; Fred Hutchinson Cancer Research Center, United States of America

## Abstract

PARP inhibition can induce anti-neoplastic effects when used as monotherapy or in combination with chemo- or radiotherapy in various tumor settings; however, the basis for the anti-metastasic activities resulting from PARP inhibition remains unknown. PARP inhibitors may also act as modulators of tumor angiogenesis. Proteomic analysis of endothelial cells revealed that vimentin, an intermediary filament involved in angiogenesis and a specific hallmark of EndoMT (endothelial to mesenchymal transition) transformation, was down-regulated following loss of PARP-1 function in endothelial cells. VE-cadherin, an endothelial marker of vascular normalization, was up-regulated in HUVEC treated with PARP inhibitors or following PARP-1 silencing; vimentin over-expression was sufficient to drive to an EndoMT phenotype. In melanoma cells, PARP inhibition reduced pro-metastatic markers, including vasculogenic mimicry. We also demonstrated that vimentin expression was sufficient to induce increased mesenchymal/pro-metastasic phenotypic changes in melanoma cells, including ILK/GSK3-β-dependent E-cadherin down-regulation, Snail1 activation and increased cell motility and migration. In a murine model of metastatic melanoma, PARP inhibition counteracted the ability of melanoma cells to metastasize to the lung. These results suggest that inhibition of PARP interferes with key metastasis-promoting processes, leading to suppression of invasion and colonization of distal organs by aggressive metastatic cells.

## Author Summary

Metastasis is the spread of malignant tumor cells from their original site to other parts of the body and is responsible for the vast majority of solid cancer-related mortality. PARP inhibitors are emerging as promising anticancer therapeutics and are currently undergoing clinical trials. It is therefore important to elucidate the mechanisms underlying the anti-tumor actions of these drugs. In our current study, we elucidated novel anti-neoplastic properties of PARP inhibitors that are responsible for the anti-metastatic effect of these drugs in the context of malignant melanoma. These effects appear to be the result of PARP-1's ability to regulate the expression of key factors, such as vimentin and VE-cadherin, involved in vascular cell dynamics and to limit pro-malignant processes such as vasculogenic mimicry and EMT.

## Introduction

Metastatic melanoma is a fatal malignancy that is remarkably resistant to treatment; however, the mechanisms regulating the transition from the primary local tumor growth to distant metastasis remain poorly understood. Metastasis, defined as the spread of malignant tumor cells from the primary tumor mass to distant sites, involves a complex series of interconnected events.

Understanding the biochemical, molecular, and cellular processes that regulate tumor metastasis is of vital importance. The metastatic cascade is thought to be initiated by a series of genetic alterations, leading to changes in cell-cell interactions that allow the dissociation of cells from the primary tumor mass. These events are followed by local invasion and migration through proteolitically modified extracellular matrix (ECM). To establish secondary metastatic deposits, the malignant cells evade host immune surveillance, arrest in the microvasculature, and extravasate from the circulation. Finally, tumor cells can invade the local ECM, proliferate, recruit new blood vessels by induction of angiogenesis, and then expand to form secondary metastatic foci [1].

Several key steps in metastatic progression involve tumor-associated endothelial cells (EC) [2]. Both angioinvasion and angiogenesis require disruption of endothelial integrity for tumor cell transmigration across the endothelium, EC migration and EC access for mitogenic stimulation. An essential step in angioinvasion and angiogenesis is the disruption of the adherent junctions between EC. Vascular endothelial cadherin (VE-cadherin; also known as cadherin 5) is the most important adhesive component of endothelial adherent junctions [3]; while ectopic expression of VE-cadherin in malignant melanoma cells confers this tumor the capability to form vessel-like structures that contributes to the lack of efficient therapeutic strategies and increases the risk of metastatic disease [4].

Epithelial-mesenchymal transition (EMT) is a trans-differentiation characterized by decreased epithelial markers such as E-cadherin[5]. EMT is a dynamic process resulting in the acquisition of cell motility with decreased adhesive ability for body organization that includes embryonic development and wound healing. Currently, EMT is thought to be a key step in the process of cancer metastasis [6]. Molecular markers of EMT include E-cadherin down-regulation, responsible for the loss of cell-cell adhesion, up-regulation of matrix-degrading proteases and mesenchymal-related proteins such as vimentin and N-cadherin, actin cytoskeleton reorganization, and up-regulation and/or nuclear translocation of transcription factors underlying the specific gene program of EMT, such as β-catenin and members of the Snail1 family [6].

The nuclear protein PARP-1, known to function as a DNA damage sensor and to play a role in various DNA repair pathways, has recently been implicated in a broad variety of cellular functions, including transcriptional regulation [7]. PARP inhibitors exhibit antitumor activity in part due to their ability to induce synthetic cell lethality in cells deficient for homologous recombination repair [8], [9], [10], [11]. PARP inhibitors also possess anti-angiogenic properties [12], [13], [14], [15], and recently, our group reported that PARP inhibition results in the down-regulation of Snail1 by accelerating the degradation of this protein [16]. In the present study we aimed to address the potential of PARP inhibition as modulators of metastasis [16].

The results presented here indicates that PARP inhibition, through down-regulation of the intermediary filament vimentin in both endothelial and melanoma cells, led to a reversion of mesenchymal phenotype in both cell types and prevented malignant melanoma cells from developing vasculogenic mimicry. As monotherapy, PARP inhibition displayed an anti-metastatic effect in a model of murine melanoma. Moreover, we identified vimentin as an upstream modulator of EMT: forced expression of vimentin was sufficient to induce tumor cell transformation through the ILK/GSK-3β signaling axis. The ability of PARP inhibition to modulate vimentin levels (and hence EMT), the interference with vasculogenic mimicry, and the modulation of endothelial plasticity allowed PARP inhibitors to exert a multifaceted antimetastatic effect to counteract the progression of malignant melanoma.

## Results

### PARP inhibition induced down-regulation of vimentin expression in endothelial cells

**Figure 1:**
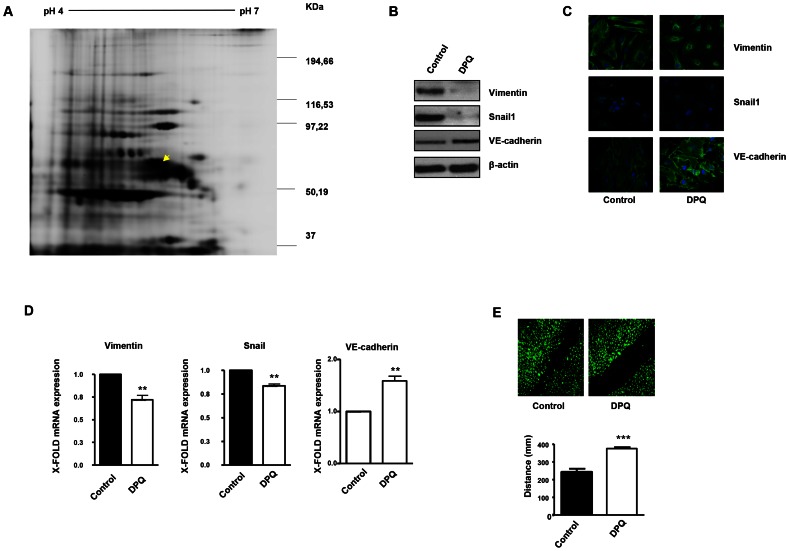
PARP inhibition down-regulates vimentin expression and inhibits endothelial-to-mesenchymal transition in HUVECs. Cell extracts from HUVEC either treated with vehicle or 40 µM DPQ were subjected to 2D electrophoresis as described in Materials and Methods. Image analysis software (DeCyder) indicated that seven proteins exhibited decreased expression in HUVEC treated with DPQ compared to untreated cells. Proteins were identified using MALDI-TOF. Spots labeled with arrows indicate proteins that were identified by mass spectrometry (see Figure 2). (**A**) The spot with the arrow is vimentin. (**B**) PARP inhibition reduced the expression of both vimentin and Snail1 and up-regulated VE-cadherin in human endothelial cells (HUVEC) as determined by immunoblotting, indirect immunofluorescence (**C**), and mRNA levels (**D**). PARP inhibition decreased HUVEC cell migration (**E**). (**P<0.01, ***P<0.001 PARP inhibitor groups *versus* DPQ).

**Figure 2:**
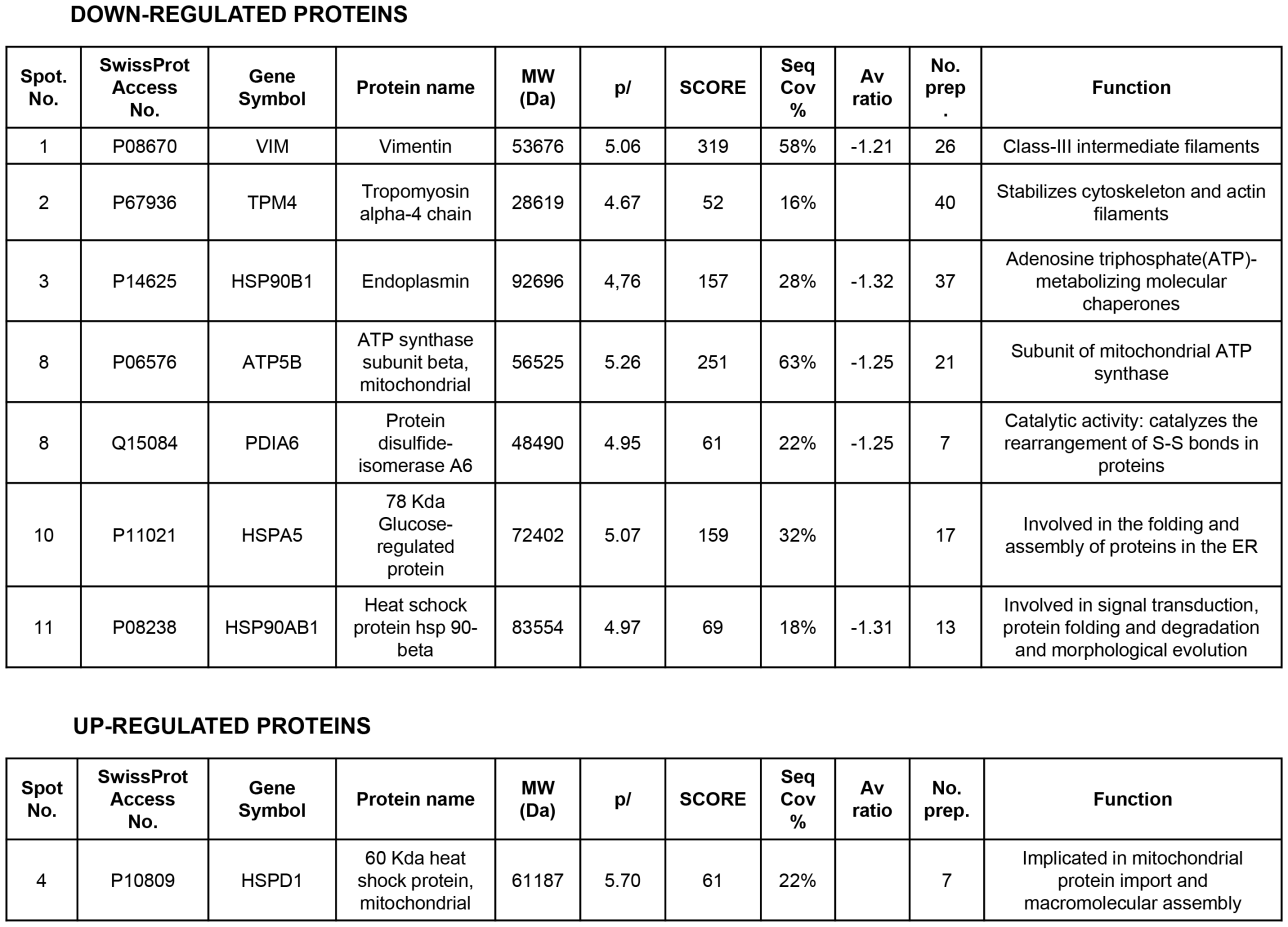
Proteins differentially expressed and identified by mass spectrometry analysis in HUVEC. The level of expression of various proteins in HUVEC was altered following PARP inhibition as determined by 2D-DIGE, and the proteins were positively identified using mass spectrometry analysis. Of particular interest for this study was vimentin, the major structural protein of intermediary filaments (spot 1). Expression of this protein was decreased in HUVEC following PARP inhibition. The proteins were identified by MALDI-TOF. Sequence coverage (%) and number of peptides were identified with  = 1% FDR (false discovery rate cut-off against decoy-concatenated randomized database). Coverage and score was determined using the MASCOT algorithm. The average ratio of protein expression between the control and cells treated with the PARP inhibitor DPQ was determined in HUVEC.

A number of reports from various laboratories, including ours, have identified a novel and unexpected effect of PARP inhibitors on angiogenesis, raising the possibility that PARP inhibitors may be useful as anti-angiogenic agents [13], [17]. In our present study, we disrupted PARP activation in HUVECs in an attempt to elucidate the mechanism by which PARP-1 influences endothelial cell dynamics. We have previously shown that PARP inhibitors reduced angiogenesis both *in vitro* and *in vivo* ([13] and Figure S1). To further characterize this effect of PARP inhibition on endothelial cell plasticity, we performed a proteomic analysis using primary HUVEC in the presence or absence of the PARP inhibitor DPQ (Figure 1A, Figure 2 and Figure S2). The expression levels of a number of proteins were altered following PARP inhibition, as detected by 2D DIGE electrophoresis (Figure S2) and mass spectrometry analysis (Figure 1A, Figure 2). A statistically significant down-regulation of vimentin (a class III intermediary filament), tropomyosin alpha-4 chain (involved in stabilizing actin filaments), endoplasmin (a molecular chaperone involved in processing and transport of secreted proteins), mitochondrial ATP synthase ATPB5, protein disulfide isomerase PDIA6, heat-shock 70 kD protein-5 (glucose-regulated protein, 78 kD), heat shock protein 90 kDa alpha (cytosolic), class B member 1, and HSP90AB1 occurred following PARP inhibition. An increase in the expression of the mitochondrial heat shock protein HSPD1 was also observed after PARP inhibition.

Due to its important role in the biology of endothelial cells, we focused our study on vimentin, the main structural protein of intermediary filaments. It has been reported that vimentin can be targeted for tumor inhibition due to its specific up-regulation in tumor vasculatures [18], [19]. To confirm the results of our proteomic analysis, we performed western blot in HUVEC either treated with DPQ (right) or left untreated. In Figures 1B and 1C, western blot and indirect immunofluorescence analysis indicated that vimentin expression was down-regulated in HUVEC cells treated with DPQ.

Figure 1B and 1D show that PARP inhibition affected not only vimentin levels but also Snail1 and VE-cadherin protein and mRNA levels.

Endothelial to mesenchymal transition (EndoMT) is a process by which endothelial cells disaggregate, change shape, and migrate into the surrounding tissue. The process of endoMT is characterized by the loss of endothelial cell markers, such as vascular endothelial VE-cadherin, and the expression of mesenchymal cell markers, such as vimentin and Snail1 [20]. Endothelial cell migration was strongly inhibited by PARP inhibition (Figure 1E). These results suggest that PARP inhibition prevented the acquisition of a mesenchymal phenotype by endothelial cells.

### Interplay between vimentin and PARP-1 modulates the expression and activity of proteins involved in EMT

**Figure 3:**
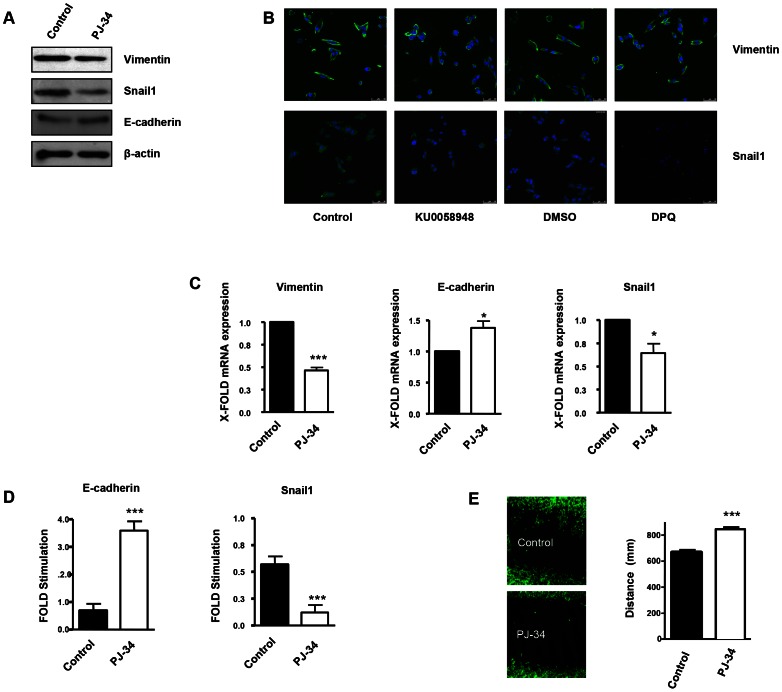
PARP inhibition inhibits the acquisition of an EMT phenotype in malignant melanoma cells. Human melanoma G361 cells and murine B16- F10 melanoma cells (Figure S3) were used for these experiments. Cells were treated with either DPQ (40 µM), PJ-34 (10 µM) or KU0058948 (100 nM) for 22 hours. IF, western blot or qPCR assays were performed to evaluate the effects of PARP inhibition on EMT markers. PARP inhibition reduced the expression of vimentin and Snail1 and up-regulated E-cadherin in human melanoma cells as determined by immunoblotting (**A**), indirect immunofluorescence (**B**), and mRNA levels (**C**). (*P<0.05, ***P<0.001, PARP Inhibitor groups *versus* the control). β-actin was used as an internal control for protein loading. (**D**) Snail1 and E-cadherin promoter activity are regulated by PARP inhibitors. Luciferase activity was determined after transfecting the constructions into G361 cells. Firefly Luciferase was standarized to the levels of Renilla Luciferase. Cells were cotransfected with 0.5 µg renilla as a transfection control and 0.5 µg of Snail1 or E-cadherin using jetPEI cationic polymer transfection reagent according to the manufacturer's instructions. Cells were compared in the presence or absence of serum (***P<0.001 control *versus* PJ-34). The expression of both Firefly and Renilla luciferase was analyzed 48 h after transfection. Cloning of the human Snail1 promoter (−869/+59) into pGL3 basic (Promega) was described previously (41). The E-Cadherin promoter was cloned into pGL3-basic (Promega) to generate pGL3-E-cadherin (−178/+92). (**E**) Inhibitory effect of PARP on B16F10 motility. Treatment with the PARP inhibitor PJ-34 (10 µM) decreased cell migration in vitro. Migration was quantified as distance between Wound Healing limits (*** *P<*0.001 control *versus* DPQ).

Vimentin is a well-known marker of EMT, which is a hallmark of primary tumor progression to a metastatic phenotype. We tested the impact of vimentin down-regulation (induced by PARP inhibition or vimentin silencing) on EMT differentiation in various melanoma cell lines and in endothelial cells. One major event induced by PARP inhibition, in the process of EMT is the up-regulation of E-cadherin expression through the inactivation of the transcription factor Snail1. Snail1 and vimentin levels were both down-regulated following PARP inhibition, indicating a disruption EMT in the absence of PARP activation (Figure 3A in G361 cells and Figure S3B in B16-F10 cells). Down-regulation of PARP activity was confirmed in G361 following H_2_O_2_ treatment as a positive control of PARP-1 activation and poly(ADP-ribose) (PAR) synthesis (Figure S4). Vimentin and Snail1 mRNA levels were decreased after PARP inhibition (Figure 3C and Figure S3C). In Figures 2B and Figure S3A, indirect immunofluorescence showed that vimentin expression was down-regulated in melanoma cells treated with DPQ or KU0058948 (G361 cells, Figure 3B) or PJ-34 (B16-F10 cells, Figure S3A). Using two different luciferase reporter plasmids under the control of a Snail1 responsive sequence and the E-cadherin promoter, we found that PARP inhibition affected negatively the activation of Snail1 and activated the expression of the E-cadherin promoter (Figure 3D and Figure S3D). Wound healing experiments also revealed decreased wound closing following treatment with a PARP inhibitor, PJ-34 (Figure 3E).

**Figure 4:**
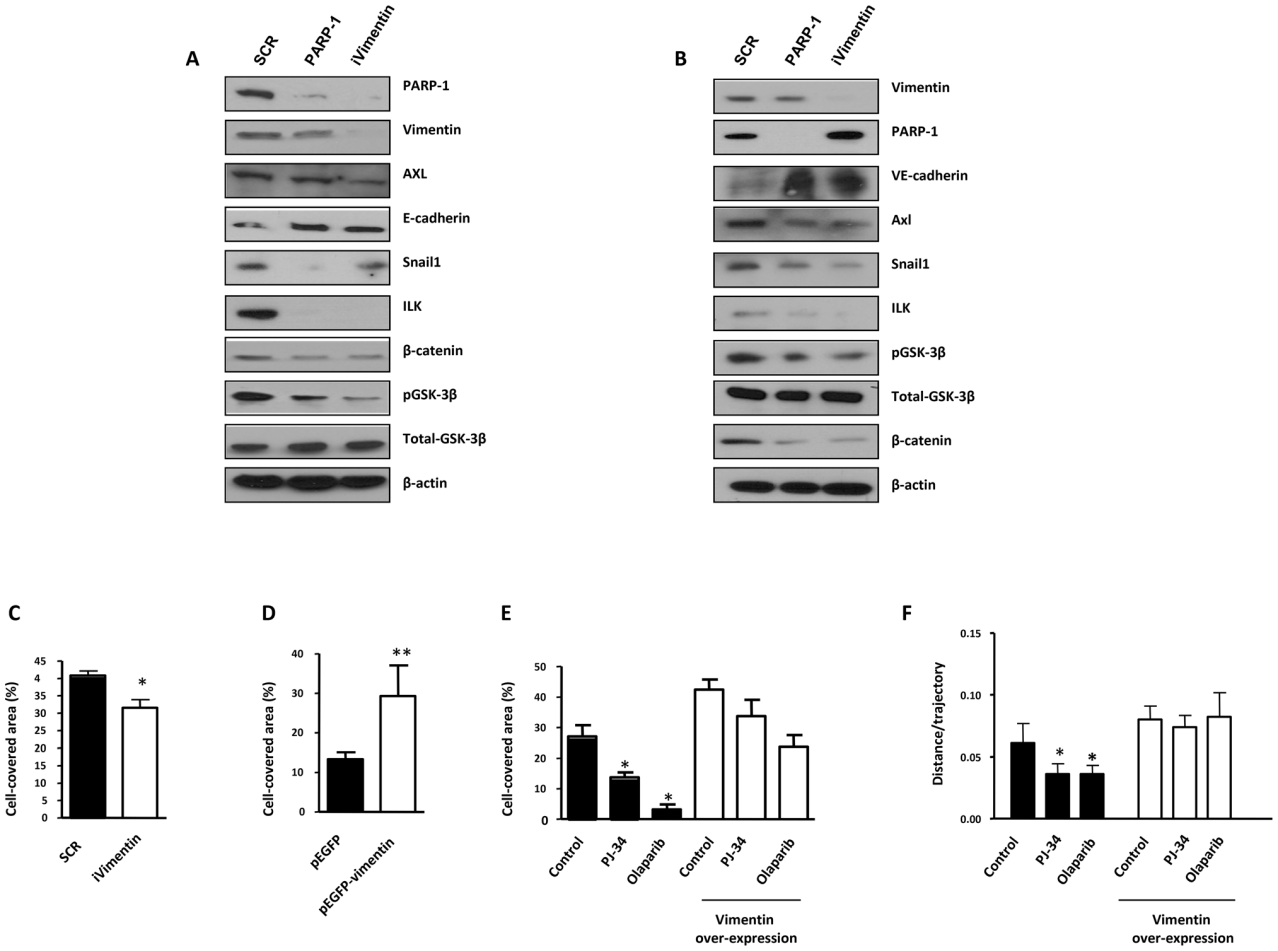
PARP-1 or vimentin is sufficient to reverse EMT and confer increased cell motility. (**A**) Melanoma (G361) and endothelial (HUVEC) (**B**) cells were silenced for PARP-1 or vimentin and the expression levels of Axl, E-/VE-cadherin, Snail1, ILK, β-catenin, GSK-3β, PARP-1, and vimentin were determined by immunoblot. (**C**) HUVEC were silenced for vimentin and wound healing was measured. After over-expression of vimentin wound healing closure was measured in HUVEC cells (**D**) or B16-F10 (**E**). (**F**) Cell migration was analyzed in epithelial cell line Madin Darby canine kidney (MDCK) cells transfected with either GFP or GFP-vimentin using video-microscopy and MetaMorph Image Analysis software. While vimentin was able to increase the length of the trajectories in the absence or presence of hepatocyte growth factor (HGF), treatment with PARP inhibitor resulted in a sustained reduction in cell motility (*P<0.05 PJ-34 or olaparib *versus* control).

We have also evaluated the effect of both PARP-1 and vimentin silencing on the expression of Axl, a key determinant of cell migration and EMT promotion [21]. Following PARP-1 silencing in HUVEC or G361 cells, the EMT marker Snail1 decreased while E or VE-cadherin were upregulated (Figure 4A and 4B respectively). Interestingly, Axl expression was also down-regulated in parallel with decreased levels of vimentin. Vimentin knockdown also caused a global alteration in the expression of EMT markers. Under these conditions, Axl levels were decreased (Figure 4A and 4B), suggesting that vimentin down-regulation was sufficient to drive tumor cells toward an epithelial state.

We next sought to determine if alterations in vimentin levels were sufficient to alter or reverse EMT progression. Vimentin is known to positively influence tumor cell migration. To test the impact of vimentin expression on cell migration and invasion we performed either silencing or over-expression in endothelial and melanoma cells. Following vimentin knockdown, wound healing closure in HUVEC cells was significantly diminished (Figure 4C) while its over-expression increased wound healing efficiency (Figure 4D). The same approach was used in B16F10 melanoma cells where over-expression of vimentin increased significantly cell migration (Figure 4E). Nonetheless, inhibition of PARP had a less impact on cell migration after vimentin over-expression, suggesting that the levels of vimentin were implicated in the effect of PARP inhibition on cell motility (Figure 4E), although a multifactorial mechanism for downstream effect of PARP inhibition could not be excluded.

To further confirm the role of vimentin in PARP-inhibitor-induced impaired cell migration we decided to analyze the effect of vimentin over-expression and PARP inhibition in a well-established model of epithelial cells, MDCK, that undergo EMT after hepatocyte growth factor (HGF) treatment, including fast movement and circularity (scattering) [22]. The trajectories of cell migration were determined under video-microscopy and analyzed using MetaMorph image analysis software. Global trajectories after expression of GFP-vimentin in the presence or absence of PARP inhibitor and HGF were determined. Treatment with the PARP inhibitor PJ-34 or olaparib resulted in decreased cell motility in cells transfected with empty GFP vector (Figure 4F). Vimentin expression increased cell motility (Figure 4F, right), and PARP inhibition was unable to prevent this increase, suggesting that vimentin down-regulation is needed for the effect of PARP inhibition in reversing the EMT phenotype.

**Figure 5:**
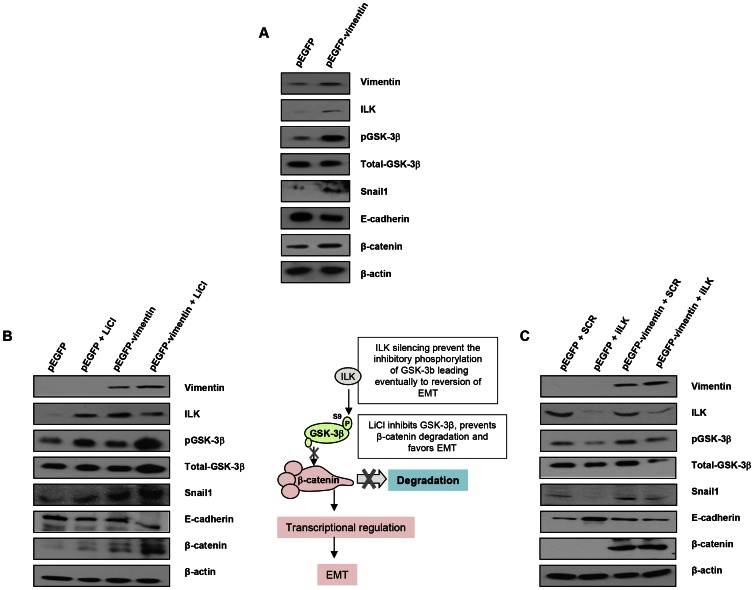
Interaction between vimentin over-expression and the activation of EMT signaling pathway. (**A**) Over-expression of vimentin in G361 cells. (**B**) Forced expression of vimentin drives human breast tumor epithelial cells (MCF7) to a mesenchymal phenotype through the integrin-linked-kinase/GSK-3β axis. 5 mM LiCl was used to inhibit GSK-3β, as detected by the accumulation of beta-catenin. (**C**) ILK was knocked down to analyze the significance of the interaction between vimentin and ILK in promoting the transition to a mesenchymal phenotype.

To characterize more in-depth the implications of vimentin expression in the context of EMT, we expressed GFP-vimentin in both a human melanoma cell line (Figure 5A) and a human breast tumor cell line with an epithelial phenotype (MCF7) (Figure 5B and 5C); MCF7 cells were chosen due to the lack endogenous vimentin expression compared with melanoma G361 cells (Figure 5A, G361 cells and Figure 5B and 5C, MCF7 cells). GFP-vimentin over-expression alone induced a mesenchymal phenotype characterized by Snail1 up-regulation, loss of E-cadherin, increased pGSK-3β (inactive form) and β-catenin expression (Figure 5A, 5B and 5C).

The most remarkable effect of PARP or vimentin silencing observed in our model was the down-regulation of ILK and GSK-3β (Figure 4A and 4B). In order to get mechanistic information on the interaction between vimentin over-expression and the activation of EMT signaling pathway, we focused in the axis ILK/GSK-3β, which plays a central role in EMT commitment, upstream of Snail1. Inhibition of GSK-3β was achieved by LiCl treatment while its activation was driven by silencing the kinase, ILK, which is the upstream inhibitory kinase for GSK-3β (Figure 5, central panel). Specifically, inhibition of GSk-3β (which was confirmed by an increase in the level of inhibitory phosphorylation of GSK-3β at Ser9) with LiCl, activated EMT and resulted in E-cadherin down-regulation, Snail1 accumulation and increased levels of β-catenin (Figure 5B); concomitantly, E-cadherin was down-regulated following GSk-3β inhibition by LiCl (Figure 5B) or exogenous expression of vimentin (Figure 5).

GSk-3β activation is achieved through the silencing of its upstream inhibitor integrin-linked kinase (ILK). ILK knockdown resulted in Snail1 down-regulation and increased E-cadherin expression (Figure 5C). Interestingly, exogenous vimentin expression completely prevented siILK-induced E-cadherin up-regulation and partially prevented the reduction of Snail1 expression. These results suggested that vimentin, when over-expressed, is sufficient to drive the phenotypic changes associated with a mesenchymal cell status, depending on the activation of GSk-3β, whose inhibition accentuated vimentin-induced changes, while its activation (following ILK-silencing), abolished vimentin-induced E-cadherin decrease and Snail1 accumulation (Figure 5C).

### PARP inhibition suppresses vasculogenic mimicry in malignant melanoma cells

**Figure 6:**
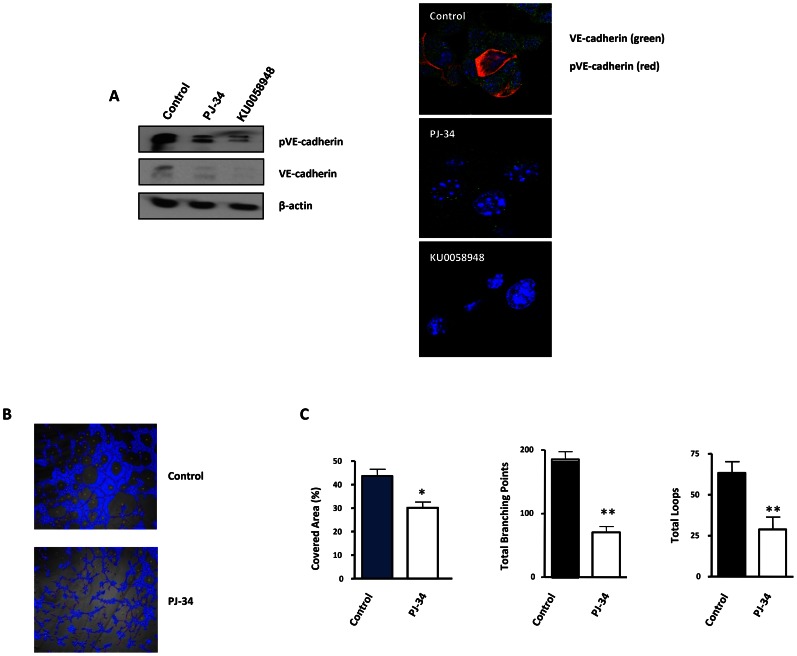
Vasculogenic mimicry is reduced by PARP inhibition in cells and in xenogafts of malignant melanoma. (**A**) Western-blot and immunofluorescence of VE-cadherin and pVE-cadherin in B16-F10 cells treated with PJ-34 or KU0058948. (**B, C**) B16-F10 cells were cultured on polystyrene-treated culture slides and treated with the PARP inhibitor PJ-34 at 20 µM or left untreated. Following treatment, pictures were taken and analyzed using Wimasis image analysis software. “Branching points”: crossroads from at least three “branches”. “Loops”: Closed areas surrounded by cells. Four independent experiments were performed (*P<0.05; **P<0.01).

The formation of patterned networks of matrix-rich tubular structures in three-dimensional culture is a defining characteristic of highly aggressive melanoma cells. It has been demonstrated that aggressive melanoma cells in which VE-cadherin was repressed, could not form vasculogenic-like networks [23], suggesting that tumor-associated misexpression of VE-cadherin (observed in melanoma cells) is instrumental in allowing endothelial cells to form vasculogenic networks. We measured VE-cadherin protein levels in B16-F10 cells after treatment with the PARP inhibitor PJ-34 or KU0058948. VE-cadherin expression was strongly down-regulated following PARP inhibition. We tried to confirm this result by indirect immunofluoresce of VE-cadherin, however the protein was barely detected, as was the case for the protein in western blot (Figure 6A). Phosphorylation of VE-cadherin has been shown to correlate with loss of function of VE-cadherin and increased vascular permeability [24], as is the case for pseudo vessels during VM. PARP inhibition was able to impact negatively on the levels of both total and phosphorylated VE-cadherin, which, indeed, had a membrane and cytoplasmic distribution (Figure 6A). The consequences for the down-regulation of both total and phosphorylated VE-cadherin by PARP inhibitors during VM are now being investigated in our laboratory.

VM was measured in vitro using B16F10 cells cultured in matrigel coated plates (Figure 6B). All markers of VM structure formation (covered area, tube length, branching points and loops) were significantly decreased after inhibition of PARP with PJ-34 (Figure 6C).

### PARP inhibition protects against lung-metastasis of murine melanoma cells

**Figure 7:**
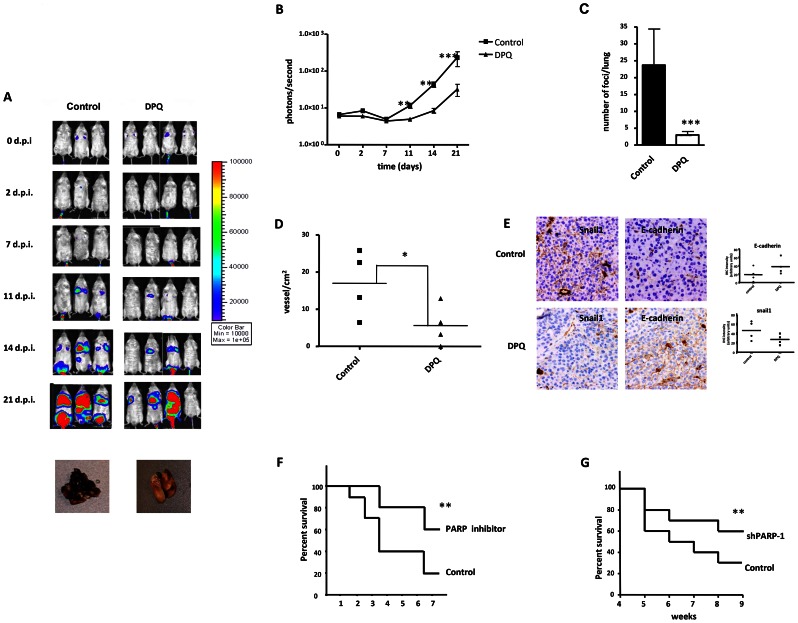
Decreased melanoma-induced lung metastasis following PARP inhibition. (**A**) Mice were inoculated with the murine melanoma cell line B16-F10-luc. Localization and the intensity of luciferase expression were monitored by *in vivo* bioluminescence imaging (dpi, days post cells injection). At the bottom of Figure A two lungs from vehicle (left) or DPQ (right) treated mice are shown. Lungs were extracted to analyze the number of melanoma foci. Quantification of luciferase activity over time shows the average light (photons) emission in photons/s (**B**) (**P<0.01; ***P<0.001 *versus* DPQ). (**C**) The number of metastatic foci/lung were counted macroscopically (***P<0.001). (**D**) Angiogenesis was measured using a specific endothelial cell marker (tomato lectin) and measured as blood vessels per mm^2^ in tumor sections of lung metastasis (Columns, mean ± SE. *P<0.05, with respect to control and DPQ–treated mice. (**E**) Immunohistochemistry staining of Snail1 and E-Cadherin in lung metastasis and quantitation using ImageJ , colour deconvolution plugin (**F and G**) Kaplan-Meyer survival curve shows the survival advantage of DPQ-treated mice following intravenous tail injection of melanoma cells as previously described in mice treated with DPQ (**F**) or injected with B16F10 stably silenced for PARP-1 (**G**) (** *P<*0. 01).

We next aimed to examine the effect of PARP inhibition on melanoma tumor growth of cells subcutaneously implanted in C57BL/6 mice. Mice were treated every two days with 15 mg/kg (i.p.) of the PARP inhibitor DPQ or vehicle. A significant difference in tumor growth was found after 14 days of tumor implantation in the DPQ-treated group compared to the control (Figure 7 and Figure S5A).

To evaluate the direct effects of the PARP inhibitor DPQ on tumor metastasis, we used a well-characterized model of experimental lung metastasis [25]. Experimental metastasis model provide several advantages for investigation. The time course for model maturity is generally rapid, the biology of metastasis is reproducible and consistent, and we control de number and type of cells that are introduced to the circulation [26]. B16-F10 cells were tail vein injected into mice, and the mice were then treated with 15 mg/kg of the PARP inhibitor DPQ or vehicle three times per week over a three-week period. Tail vein injection results primarily in pulmonary metastases. Photon emission was acquired every two days. Seven days after B16-F10 cell injection, a photon signal was already detected in the lungs (Figure 7B), and DPQ treatment significantly suppressed lung metastasis compared to the control throughout the duration of the experiment (21 days). Similar results were obtained using the clinically relevant PARP inhibitor olaparib (Figure S6). Metastatic foci were also detected in other organs upon mice autopsy. These organs included the liver, kidney, spleen, gut, stomach and heart (Figure S5B). In all cases, the incidence of metastatic foci was reduced compared to lung metastasis. DPQ-treated mice exhibited a decreased incidence of extra-pulmonary metastasis compared to the control. Pathologic analysis of the lungs showed a decrease in size and number of metastatic foci (more than 80%) after DPQ treatment (Figure 7C) that was accompanied by a reduced number of tumor vessels in both primary subcutaneous tumors and lung metastasis (Figure 7D), suggesting that the anti-angiogenic effect of PARP inhibition may be involved in the observed reduction in metastatic progression. Apoptotic and mitotic rate were not significantly different in tumors derived from DPQ-treated or untreated mice (Figure S7). To investigate *in vivo* the effect of PARP inhibition on the expression of Snail1 and E-cadherin, we performed immunohistochemistry for these EMT markers in metastatic lung tumors (Figure 7E). We observed that Snail1 was highly expressed in the vessels of tumors derived from the untreated group. This expression exhibited both nuclear and cytoplasmic distribution as previously reported [27]. Metastatic lung tumors derived from DPQ-treated mice displayed reduced expression of Snail1 as well as an increase in E-cadherin expression, similar to the results obtained in cultured melanoma cells. These data indicate that the *in vivo* expression of EMT markers within tumors is also reduced following treatment with PARP inhibitor. We also performed a Kaplan Meyer curve to compare the mortality of both groups of mice, and we observed a statistically significant difference in the survival rate from <4 weeks in the untreated group to >8 weeks in the DPQ-treated mice (Figure 7F). Survival of mice injected with B16-F10 cells stably expressing shRNA targeting PARP-1 (Figure 7G), was also significantly increase.

### Human melanoma tissue array

To determine the correlation between PARP-1 expression and disease progression in human melanoma, we used IHC to analyze the levels of vimentin, PARP-1, Snail1, E-cadherin and MITF in nodular and metastatic melanoma frozen biopsies. Vimentin was expressed in all biopsies derived from both nodular and metastatic melanoma; however, the level of expression was elevated in nodular melanoma, which is the initial stage of the disease. PARP-1 expression was positively correlated with vimentin expression, suggesting an association between the *in vivo* expression of both proteins (Figure S8, Table S1). Expression of the Snail1 and microphthalmia-associated transcription factor (MITF), which is a melanocyte marker, is also increased in metastatic melanoma. Interestingly, nodular melanoma did not express Snail1 while 40% of metastatic melanoma samples displayed Snail1 expression. Loss or reduction of E-cadherin and increased expression of EMT markers is frequently associated with the development of an invasive phenotype in cancer. Expression of E-cadherin in normal melanocytes is significantly reduced during the initial steps of melanoma progression [28]; however, elevated levels of E-cadherin are found at advanced stages of the disease [29]. E-cadherin expression was similar in both nodular and metastatic melanoma (Table S1), which is in agreement with previous publications. These findings suggest that in human melanoma, there is a complex interconnection between the expression levels of various disease markers and the expression of PARP-1, although we have detected a strong correlation between vimentin and PARP-1 expression (Figure S8).

## Discussion

PARP inhibitors are a novel and important class of anticancer drugs, and there are now more than 40 clinical trials that are ongoing or in development to study the effectiveness of PARP inhibitors in the treatment of various cancers. Given the enormous interest in this target, it is important to understand the underlying mechanisms by which PARP-1 and other PARPs function in tumor cell biology. Until recently, the development of PARP-1 inhibitors has focused almost exclusively on the function of this enzyme in DNA repair. Emerging literature, however, indicates other activities of PARP-1 that may explain the *in vivo* potency of some PARP-1 inhibitors that cannot be entirely attributed to their apparent *in vitro* activity and that could provide additional targets for anti-cancer therapies. In addition to its direct role in DNA-damage recognition and repair, PARP-1 can regulate the function of several transcription factors, including p53 and NF-κB. In the context of certain cancers, PARP-1 interacts with the transcription factors HIF1 [13] and Snail1 [16]. The mechanisms underlying the effects of PARP inhibition on vascular plasticity and metastasis remain relatively unknown. Our current study identifies PARP-1 as a pivotal modulator of the molecular and functional changes characteristic of EndoMT (involved in the loss of function of tumor-associated vessels) and of the phenotypic switch that facilitates the acquisition of pro-metastatic capacities by tumor cells. Proteomic analysis of endothelial cells that have been treated with a PARP inhibitor identifies the intermediary filament protein vimentin as a target of PARP inhibition. Intermediary filaments such as vimentin and keratins are known to play non-mechanical roles in protein trafficking and signaling (reviewed in [30]), which in turn influence cellular processes such as cell adhesion and polarization. Vimentin is abundantly expressed by mesenchymal cells and plays a critical role in wound healing, angiogenesis and cancer growth. Vimentin has also been described as a tumor-specific angiogenesis marker, and targeting endothelial vimentin in a mouse tumor model significantly inhibited tumor growth and reduced microvessel density [31].

Vimentin is both an EMT and EndoMT marker and is also over-expressed in tumor samples compared to normal tissues. This protein also contributes to tumor phenotype and invasiveness [18], [19]. Our findings indicate that PARP inhibitors reduce the metastatic potential of melanoma cells, at least in part, through their ability to down-regulate vimentin expression.

Vimentin expression has been shown to be transactivated by β-catenin/TCF and thus increasing the tumor cell invasive potential [19]. It has been shown that NF-κB, a key protein regulating the immune and inflammatory process, also plays an important role in regulating EMT process and its inhibition in the mesenchymal cells reversed the EMT process, suggesting the importance of NF-κB in both activation and maintenance of EMT [32]. Since vimentin is over expressed during EMT process, and NF-κB being one of the transcription factors binding to vimentin promoter, it would be tempting to speculate that this over-expression of vimentin is a result of activated NF-κB in cancer cells. Also, TGFβ1 response element was found within the activated protein complex-1 region of the vimentin promoter and was involved in regulation of vimentin expression in myoblasts and myotubes [33]. Interestingly, ADP-ribosylation of Smad proteins by PARP-1 has been shown to be a key step in controlling the strength and duration of Smad-mediated transcription [34]. Regulation of vimentin levels by PARP inhibition may also involve other transcription factor such as Snail1 and HIF-1/2.

Our results also reveal that vimentin levels are not merely a hallmark of EMT. While silencing of vimentin in melanoma cells can reverse the EMT phenotype, in part by promoting down-regulation of the protein kinase Axl that is involved in cell motility, forced expression of vimentin in tumor cells lacking this protein is sufficient to trigger the switch from epithelial to mesenchymal phenotype. GSK-3β is an upstream regulator of key factors involved in EMT such as Snail1 and β–catenin. We hypothesized that vimentin may be involved in the modulation of this upstream regulator of EMT. Indeed, vimentin expression potentiated LiCl-(a GSK-3β inhibitor) induced EMT (Figure 5B) and counteracted the inhibitory action of ILK-silencing (leading to GSK-3β activation) in the context of EMT (Figure 5C). Mechanical signals can inactivate GSK-3β resulting in stabilization of β-catenin. Intermediate filaments are important in allowing individual cells, tissues and organs to cope with various types of stress, and they play a significant role in the mechanical behavior of cells [35]. It is possible that the signaling pathway that integrates PARP activation with altered vimentin expression and fluctuations in GSk-3β activity could be related to the capability of PARP inhibitors to inactivate AKT signaling [36], which would result in GSk-3β activation and the modulation of its downstream signaling, ultimately resulting in the reversal of EMT.

Vasculogenic mimicry, as a de novo tumor microcirculation pattern, differs from classically described endothelium-dependent angiogenesis. This is a unique process characteristic of highly aggressive melanoma cells found to express genes previously thought to be exclusively associated with endothelial cells and is characteristic of aggressive melanoma tumor cells. HIF-1α and HIF-2α, transcription factors that are stabilized during conditions of oxygen depletion (hypoxia), are the master regulators of VE-cadherin. HIF-mediated transcriptional regulation during hypoxia is critical as this process induces genes that are essential for tumor cell adaptation to the stress of oxygen depletion. As a result, the expression of HIF target genes is associated with increased malignancy. Although the expression of VE-cadherin is not hypoxia-regulated, HIF-2α, but not HIF-1α, activates the VE-cadherin promoter by binding to the HRE during normoxic conditions [37]. HIF-2α expression is associated with developing endothelium, proper vascular development and increased tumor malignancy [38], [39], raising the possibility that it may be an important protein that functions in the induction of tumor cell plasticity.

Using a mouse model of melanoma lung metastasis, we also present *in vivo* evidence indicating that targeting PARP strongly reduces metastatic dissemination of melanoma cells, at least in part through inducing a reduction in tumor microvessel density along with changes in the expression pattern of EMT markers (Snail1, vimentin and E-cadherin) within the tumor.

Snail1 is a master regulator of EMT, and the activation of this protein can mediate tumor invasiveness through the transcriptional repression of E-cadherin expression. Regulating the activity of E-cadherin repressors represents a potentially beneficial strategy to fight cancer progression, and PARP-1 inhibitors accomplish this function by interfering with Snail1 activation.

Results from human tissue arrays of melanoma suggest a complex interaction between PARP-1 expression and melanoma progression. It is difficult to verify EMT experimentally *in vivo* due to the reversible and dynamic nature of the process. Although melanoma cells are not epithelial in nature, the EMT for this tumor is well characterized and the relevance of the cadherin switch has been previously described using several experimental approaches, demonstrating that melanoma cell lines transfected with N-cadherin are morphologically transformed from an epithelial-like shape to a fibroblast-like shape [37]. Adenoviral re-expression of E-cadherin in melanoma cells down-regulates endogenous N-cadherin and reduces the malignant potential of these cells [37].

**Figure 8:**
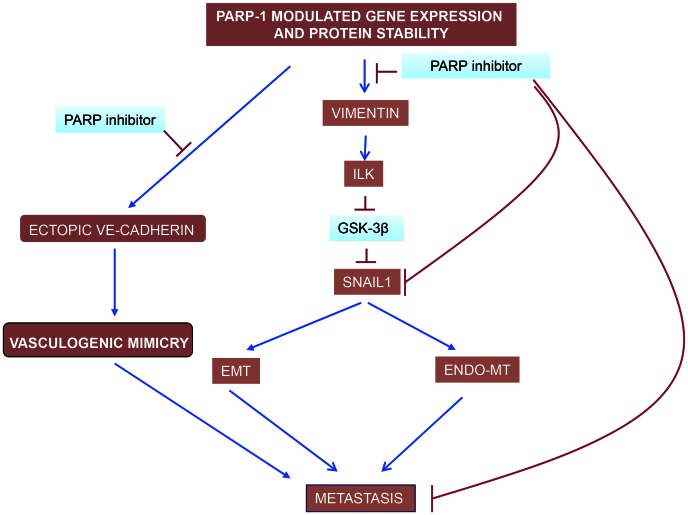
PARP inhibitors interfere with EndoMT, EMT and vasculogenic mimicry in melanoma cells. Vimentin down-regulation is pivotal in driving this effect of PARP inhibitors, acting through the ILK/GSk-3β (see the text). While VE-cadherin is upregulated by PARP inhibitors in endothelial cells, contributing to vascular normalisation, its levels are down-regulated in malignant melanoma cells (Figure 5C). The ultimate reason for this cell-specific regulation of VE-cadherin expression by PARP is being studying currently in our laboratory.

Globally, our study shows that PARP inhibition is perturbing metastatic transformation at least at three levels (Figure 8): i) decreasing abnormal tumor angiogenesis through its ability to counteract Endo-MT; ii) preventing from acquisition of EMT and iii) limiting vasculogenic mimicry in melanoma cells.

Over the past few years, PARP has emerged as a strong and effective target for first line anticancer therapy. Due to its ability to regulate a number of cellular functions (from DNA repair to cell death and transcription), inhibition of PARP may affect multiple facets of tumor metabolism. These findings strongly indicate that several novel activities of PARP-1 may contribute to the effects of anti-cancer therapy targeting this protein by interfering with tumor physiology and the tumor microenvironment. Given these findings, it is of vital importance that we elucidate mechanisms regulating novel functions of PARP-1 and poly (ADP-ribose) in tumor biology so that PARP inhibitors can ultimately make the transition to routine clinical use.

## Materials and Methods

### Cell culture

Human umbilical vein endothelial cells (HUVEC) were cultured in EGM-2 Endothelial Cell Growth Medium-2 (LONZA). Cells were subjected to experimental procedures within passages 3–6. B16-F10-luc-G5 cells stably expressing plasmids pGL3 control (SV40-luc) (Promega) and pSV40/Zeo (Invitrogen). Human (G361), murine (B16-F10) malignant melanoma cells and breast cancer (MCF7) cells were cultured in DMEM containing 10% fetal bovine serum, 0.5% gentamicin (Sigma, St. Louis, MO), and 4.5% glucose. All cells were cultured at 37°C (5% CO2). The tumor cell lines have been developed as described in detail previously [40]. Melnikova et al. [41] found that unlike human melanomas, the murine melanomas cell lines did not have activating mutations in the Braf oncogene at exon 11 or 15. All of the cell lines also expressed PTEN protein, indicating that loss of PTEN is not involved in the development of murine melanomas. This B16-F10 cell has previously been shown to be sensitive to stable depletion of PARP-1 in vivo melanoma growth [17]. Previous publication from our lab in G361 cells show similar results [16]. Cells were treated with the PARP inhibitors 3,4-dihydro-5-[4-(1-piperidinyl)butoxyl]-1(2H)-isoquinolinone (DPQ), [N-(6-Oxo-5,6-dihydro-phenanthridin-2-yl)-N,N-dimethylacetamide] (PJ-34) (Alexis Biochemicals, San Diego, CA) (as described [42], KU0058948 (as we shown in previous publications [16] or Olaparib (KU0059436, Selleckchem) for 22 hours. For capillary-like formation assays, 25 µL of Matrigel (BD Biosciences) were spread onto eight-chamber BD Falcon glass culture slides (BD Biosciences) or onto 96-well plates. Cells were seeded at 2.5×10^4^ cells per well (high density) in eight-chamber slides and at 5×10^3^ cells per well (low density) in 96-well plates and maintained in RPMI supplemented with 1% FBS [13].

### Western blot, quantitative RT-PCR and luciferase reporter gene assay

These assays were performed according to previously published methods [13]. Primary antibodies used in these studies consisted of vimentin and VE-cadherin (mouse monoclonal), E-cadherin (rabbit polyclonal) (Santa Cruz Biotechnology), Snail1 and pVE-cadherin (rabbit polyclonal) (Abcam), ILK (rabbit monoclonal) (Millipore), Axl (rabbit polyclonal), total-GSK-3β (mouse monoclonal) and pGSK-3β (rabbit monoclonal) (Cell Signaling), β-catenin (mouse monoclonal) (BD Transduction Laboratories), PARP-1 (monoclonal) (Alexis) as well as β-actin (Sigma Aldrich). Quantitation of western blots was performed using Quantity One software analysis and all densitometries were normalized for loading control (Table S2).

Luciferase activity was determined after transfecting the constructions into the B16-F10 cells. Firefly Luciferase was standardized to the value of Renilla Luciferase. Cells were co-transfected with 0.5 µg renilla as control of transfection together with 0.5 µg of the Snail or E-cadherin plasmid using jetPEI cationic polymer transfection reagent according to the manufacturer's instructions. The expression of Firefly and Renilla luciferases was analysed 48 h after transfection, according of the manufacturer's instructions. Cloning of the human Snail1 promoter (−869/+59) in pGL3 basic (Promega), was described previously [43]. E-cadherin promoter were cloned into pGL3-basic (Promega) to generate pGL3-E-cadherin (−178/+92).

### Transfection of small interfering RNA

HUVEC or G361 cells were transiently transfected with an irrelevant siRNA [44], PARP-1 siRNA or vimentin siRNA (Thermo Scientific) for 24 h using JetPrime (Polyplus transfection) according to the manufacturer's recommendations. At 48 h post-transfection, the expression of PARP-1, vimentin, Axl, E-cadherin, Snail1, ILK, β-catenin, pGSK-3β and total-GSK-3β was measured. Cells were washed twice in phosphate-buffered saline (PBS) and scraped in Laemmli buffer (1 M Tris, 20% SDS and 10% glycerol) and sonicated. The protein concentration was determined using the Lowry assay. Levels of β-actin were monitored as a loading control.

### Transfection with GFP-vimentin

We used the GFP-vimentin expression vector supplied by Dr. Goldman (Department of Cell and Molecular Biology, Chicago, Illinois). For transfection, JetPrime was used according to the manufacturer's protocol.

24 h post-tranfection, 5 µM of LiCl (Sigma Aldrich) was added in MCF7 cells and 48 hours later, the expression of vimentin, ILK, pGSK-3β, total-GSk-3β, E-cadherin, Snail1 and β-catenin was measured. In other experiment, co-transfection of GFP-vimentin and ILK siRNA (Sigma Aldrich) was used the according of the manufacturer's protocol. GFP and an irrelevant siRNA [44] were used as a control.

### Migration/invasion assays

HUVEC and B16-F10 cells were cultured on coverslips in six-well cell culture dishes. Monolayer cultures were stained with CellTracker Green CMFDA in HUVEC cell (5-chloromethylfluorescein diacetate) (Invitrogen) according to manufacturer recommendations or with 4′,6′-diamidino-2-phenylindole dihydrochloride (DAPI) (post-fixation). A wound was induced in the confluent monolayer cultures, and the cultures were then treated with the indicated inhibitor. The cells were fixed with 3.7% buffered formaldehyde and then prepared for immunofluorescence. Images were captured using a confocal microscope (LEICA TCS SP5 Argon Laser 488 nm, HeNe Laser 543 nm) when the cells were stained with CellTracker Green CMFDA Abs [522 nm] and Em [529 nm] and Zeiss Axio Imager A1 microscopy for cells stained with DAPI.

The method used to Wound Healing using a service provided by Wimasis with permits users to upload their images online at any time and form anywhere and allows their images to be analyzed and the results uploaded back to the researcher's serve.

### Scattering assay

Madin-Darby canine kidney (MDCK) cells (1,5×10^4^) were seeded in 12-well tissue culture dish. After 24 h, cells were transfected with GFP or GFP-vimentin and 1 day after, cells were incubated with HGF (hepatocyte growth factor, Sigma Aldrich) or PBS. HGF is a mitogenic growth factor that is well known to induce the dissociation of islands of cells into individual cells, termed “cell scattering” or EMT. When inhibitors were used, cells were preincubated with PARP-1 inhibitor, PJ-34 or Olaparib for 2 h before addition of HGF. After 48 h, representative photographs were taken at 10× magnification using a Leica Spectral confocal laser microscope. The results were analyzed using the MetaMorph image analysis software.

### In vitro angiogenesis assay

The effect of PARP inhibitors on the formation of tube-like structures in Matrigel (BD Biosciences) was determined according to manufacturer instructions. Briefly, 24-well plates were coated with 100 µl of BD Matrigel™ Basement Membrane Matrix and allowed to solidify at 37°C in 5% CO_2_. Cells were treated with DPQ (40 µM) or PJ-34 (10 µM). After 22 h of incubation at 37°C in 5% CO_2_, the cells were fixed with 3.7% formaldehyde, and images were acquired using an Olympus CKX41 microscope. The formation of tube-like structures was then quantified. Each treatment was performed in triplicate, and the experiment was independently repeated at least three times.

### Matrigel angiogenesis assay *in vivo*

C57BL/6 mice background (8 weeks old) were subcutaneously (s.c.) flank-injected with 600 µl of matrigel (BD Biosciences) supplemented with VEGF (100 ng/ml) (Peprotech) and heparin (Sigma, 19 U). The negative controls contained heparin alone. Each group consisted of four animals. After seven days, mice were sacrificed and matrigel plugs were extracted. The angiogenic response was evaluated by macroscopic analysis of the plug at autopsy and by measurement of the hemoglobin (Hb) content within the pellet of matrigel. Hb was mechanically extracted from pellets reconstituted in water and measured using the Drabkin (Sigma-Aldrich) method by spectrophotometric analysis at 540 nm. The values were expressed as optical density (OD)/100 mg of matrigel.

### *In vivo* bioluminescence assay

This study was performed in strict accordance with the recommendations in the Guide for the Care and Use of Laboratory Animals of the Bioethical Committee of CSIC. The protocol was approved by the Committee on the Ethics of Animal Experiments of the CSIC. All surgery was performed under isoflurano anesthesia, and every effort was made to minimize suffering.

Eight-week-old male C57BL/6 albino mice (The Jackson Laboratories, Bar Harbor, MN, USA) were injected subcutaneously with B16-F10-luc-G5 cells (1×10^5^) and intravenously with B16-F10-luc-G5 cells (1×10^5^ or 5×10^5^). Three times per week mice were injected intraperitoneally with DPQ dissolved in phosphate-buffered saline/10% DMSO at a dose of 15 mg/kg body weight or olaparib at 50 mg/kg. Mice were injected intraperitoneally with D-luciferin solution dissolved in phosphate-buffered saline at a dose of 150 mg/kg body weight. After 5 to 8 minutes, the animals were anesthetized in the dark chamber using 3% isoflurane in air at 1.5 L/min and O_2_ at 0.2 L/min/mouse, and animals were imaged in a chamber connected to a camera (IVIS, Xenogen, Alameda, CA). Exposure time was 3 min in *large binning*, and the quantification of light emission was performed in photons/second using Living Image software (Xenogen). Tumor growth was monitored at 0, 2, 7, 14 and 21 days by *in vivo* imaging and bioluminiscence measurement. After 21 days, mice were sacrificed, and their organs were removed and stored in buffered formalin (3.7%) until histological staining.

### Indirect immunofluorescence

Immunostaining for vimentin, VE-cadherin, pVE-cadherin, Snail1 and E-Cadherin was performed on cells plated onto coverslips and grown for 22 h prior to experimental treatments. The culture medium was removed, and the cells were fixed (Paraformaldehyde 3%, Sucrose 2% in PBS) for 10 minutes at room temperature. Permeabilization was performed using 0.2% Triton X-100 in PBS. The coverslips were rinsed three times in PBS prior to incubation with primary antibody for 1 h at RT and then rinsed three times in PBS before incubation with the secondary antibody. Secondary antibodies were FITC-conjugated anti-mouse IgG or anti-rabbit (Sigma, St. Louis, MO). Antibodies were diluted in PBS containing 2% bovine serum albumin. Nuclear counterstaining with 4′,6′-diamidino-2-phenylindole dihydrochloride (DAPI) was performed after removal of excess secondary antibody. Slides were prepared using Vectashield mounting medium (Vector Lab., Inc., Burlingame, CA 94010), cover slipped and stored in the dark at 4°C. Immunofluorescence images were obtained in the linear range of detection to avoid signal saturation using a fluorescent microscope (Zeiss Axio Imager A1) or confocal microscopy (Leica SP5).

### Histological techniques

For conventional morphology, three buffered 4% formaldehyde-fixed, paraffin-embedded skin longitudinal tissue sections were stained with periodic acid schiff (PAS) at the end of treatment. The study was done in blinded fashion on 4-µm sections with light microscopy. The mitosis and apoptosis cells were assessed by examining their number in ten high power field (hpf) at 600× magnifications. The results were expressed as number of cells per mm^2^. For evaluation of blood vessels density, tissue sections of different groups were dewaxed, hydrated, and heat-treated in 0.01 M citrate buffer for antigenic unmasking. The rest of the procedure was carried out using an automatic immunostainer (Autostainer480, Labvision, Fremont CA, USA). The incubation time with lectin Ulex europaeus biotin conjugated was 60 min, the dilution was 1∶200, and the streptavidin-biotin-peroxidase method (Master Diagnóstica, Granada, Spain) with diaminobezidine was used as visualization system. A millimeter scale in the eyepiece of a microscope BH2 (Olympus) with 40× objective was used to count the vessel per mm^2^ of tissue section. The morphological and immunohistochemistry study was done in a double-blinded fashion by two pathologists.

### Statistical analysis

For data shown in Figure 7 and FigureS7 we have fitted the values of the average number of tumors per mouse during carcinogenesis treatment using the Mann-Whitney u-test. Statistical analysis of other experiments used unpaired Student's t-test.

## Supporting Information

Figure S1 PARP inhibitors decrease VEGF-induced tube formation in HUVECs *in vitro* and *in vivo*. Cells were collected and seeded in Matrigel-coated 48-well plates and then incubated in the absence (Control) or presence of VEGF and DPQ (40 µM) or PJ34 (20 µM). After 48 h, the morphological changes of the cells and any tubes formed were observed and recorded under a microscope. Micrographs were taken 40×. The number of tube was counted (A) (n = 4), and mean is shown. Bars ± SEM (**P<0.01 *versus* control). After subcutaneous matrigel injection in the presence and absence of PARP inhibitor DPQ, a decreased in VEGF-induced in vivo angiogenesis was observed. The formation of vessel *in vivo* was assessed after injection of HUVEC with matrigel plug contains VEGF and heparin. The neovascularizaton was evaluated by measurement of HB content of matrigel plug. The histogram represents the mean (n = 4) of the content, expressed as absorbance (DO)/100 mg of matrigel plug (B).
(TIF)

Figure S2 2D-DIGE (Differential In-Gel Electrophoresis). HUVEC were solubilized in 2D-DIGE sample buffer (40 mM Tris, 7 M Urea, 2 M Thiourea, 1% ASB-14), sonicated and then the concentration was determined using the RC hDC Protein Assay (Bio- Rad). Fifty µg of protein was then labelled with 400 pmol of CyDye DIGE Fluor minimal dyes (GE Healthcare) and incubated on ice in the dark for at least 30 min according to manufacturer instructions Cy3 (A), Cy5 (B) for samples and Cy2 (C) for internal control consisting of equal parts of all samples). The reaction was halted by the addition of 10 mM lysine and incubated on ice for 10 min. Samples were loaded onto IPG strips (7 cm, pH 4–7) (Bio-Rad) by passive rehydration for 15 h and subjected to isoelectrofocusing using the PROTEAN IEF Cell System (Bio-Rad) according to the manufacturer's protocols. For the second dimension, strips were loaded on top of 7.5% polyacrylamide gels at 150 V for 1 h. The 2D gels were then scanned using a Typhoon Imager (GE Healthcare) at 100 µm resolution with λex/λem of 488/520, 532/580, and 633/670 nm for Cy2, Cy3, and Cy5, respectively. Image analysis was performed using DeCyder 6.5 software (GE Healthcare) as described in the user manual. Six independent experiments were performed for each experimental setup. Briefly, the differential in-gel analysis (DIA) module was used for spot detection, spot volume quantification and volume ratio normalization of different samples in the same gel (D). Differentially expressed spots were considered for identification based upon the fold change (>1.1) and the t-test (*P<0.05). (E) The Image analysis DeCyder Sofware indicated those differential spots detected in HUVEC treated with DPQ cells that were subsequently identified.
(TIF)

Figure S3 PARP inhibition reduced the expression of Vimentin and Snail1 and up-regulates E-cadherin murine melanoma cells. Cells were treated with either of the PARP inhibitors DPQ (40 µM) (not shown), PJ34 at 10 µM or KU0058948 (100 nM) during 22 hours. IF (A), western-blot (B) or qPCR (C) were performed to evaluate the impact of PARP inhibition on EMT markers. *P<0.05, ***P<0.001 PARP Inhibitor groups *versus* the control. β-actin was used as internal controls for protein loading. Luciferase activity (D) was determined after transfecting the constructions into the B16-F10 cells. *P<0.05 control *versus* DPQ. The expression of Firefly and Renilla luciferases was analyzed 48 h after transfection, according of the manufacturer's instructions.
(TIF)

Figure S4 Western-blot (A) and immunofluorescence (B) of PARP activity inhibition in G361cells treated with the PARP-1 inhibitor, DPQ.
(TIF)

Figure S5 Development of subcutaneous xenografts of melanoma is reduced by treatment with the PARP inhibitor DPQ. (A) C57BL/6 albino mice (Jackson Laboratories, Philadelphia, USA), were inoculated with B16-F10-luc cells as explained in Methods. Localization and intensity of luciferase expression was monitored by in vivo bioluminescence imaging. Quantitation of luciferase activity over time in photons/s, is represented in the color bar. Vehicle (n = 4), DPQ (n = 4). **P<0.01; ***P<0.001. (B) Ex-vivo photon emission: treatment with the PARP inhibitor DPQ reduced lung and extra-pulmonary melanoma-induced metastasis.
(PDF)

Figure S6 Treatment with the PARP inhibitor olaparib decreased metastatic spread of melanoma cells. C57BL/6 mice were inoculated with B16-F10-luc cells a treated with the PARP inhibitor olaparib (50 mg/kg) as explained in Methods. Results obtained on the 17^th^ day are shown for quantitation. *P<0.05 olaparib *versus* control using the Mann-Whitney u-test.
(TIF)

Figure S7 Cell proliferation and apoptosis are not affected by PARP inhibition in metastasis. Mitosis and apoptosis were evaluated in histological metastasis slides using morphological criteria and evaluated in a blind observation by two different pathologists.
(TIF)

Figure S8 IHC evaluation of PARP-1 and EMT markers in human melanoma tissue array. Expression of PARP-1 and EMT markers in nodular and metastatic human melanoma. PARP-1 expression correlates with vimentin in nodular and metastatic melanoma. Snail1 and E-cadherin expression do not correlate with PARP-1 positivity.
(TIF)

Table S1 Results for all the different markers are presented. Samples were analyzed in a blind fashion by two different pathologists.
(TIF)

Table S2 Quantitation of western-blots. All western blots shown have been quantified using Quantity One software from Bio-Rad.
(PDF)
