# Supplementary figures and images for: PARP-1 Regulates Metastatic Melanoma through Modulation of Vimentin-induced Malignant Transformation
Source: PLoS Genet. 2013 Jun 13;9(6):e1003531. doi: 10.1371/journal.pgen.1003531 (PMC3681683; doi:10.1371/journal.pgen.1003531)

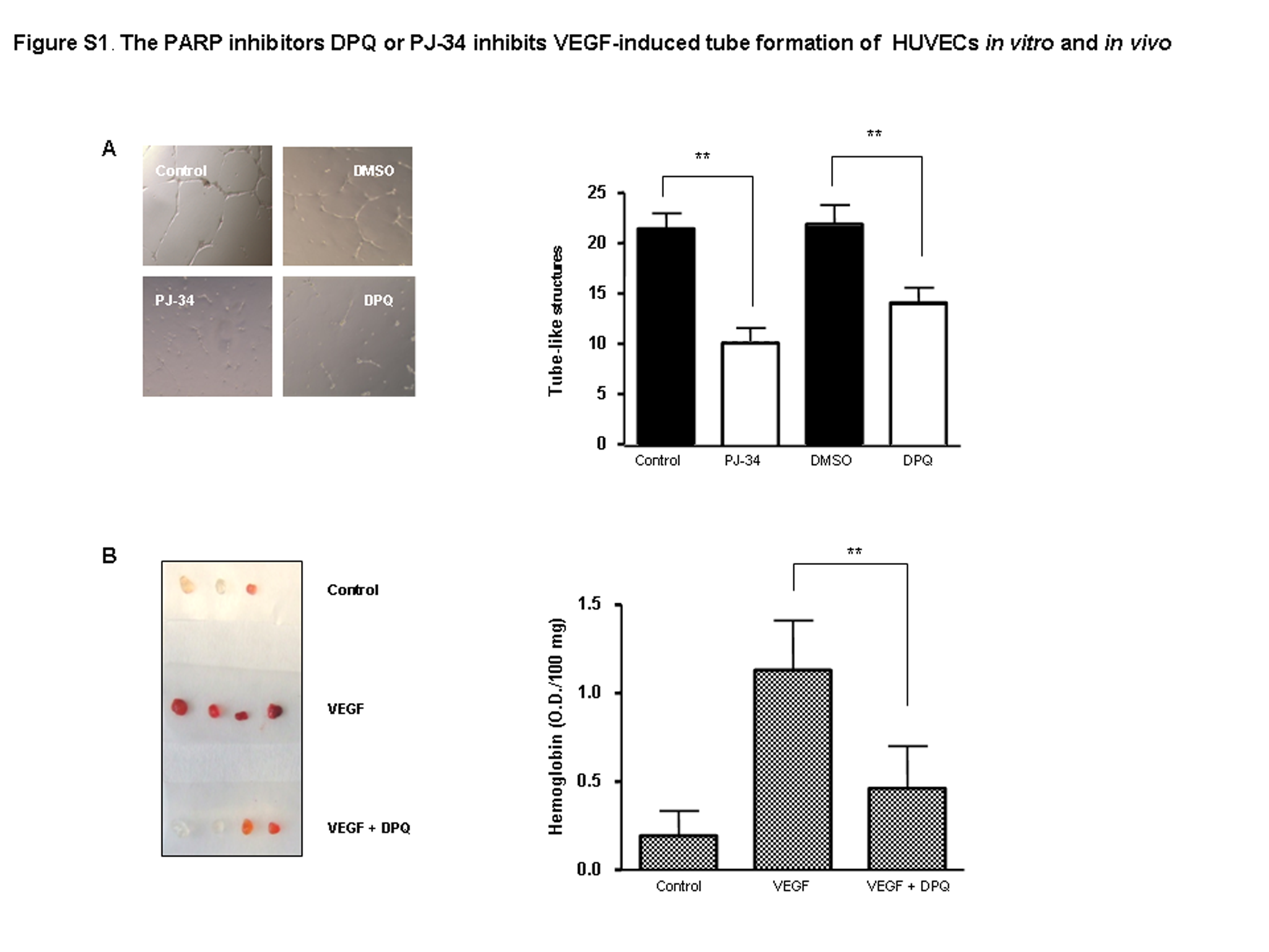

Supplement: Figure S1 — PARP inhibitors decrease VEGF-induced tube formation in HUVECs in vitro and in vivo. Cells were collected and seeded in Matrigel-coated 48-well plates and then incubated in the absence (Control) or presence of VEGF and DPQ (40 µM) or PJ34 (20 µM). After 48 h, the morphological changes of the cells and any tubes formed were observed and recorded under a microscope. Micrographs were taken 40×. The number of tube was counted (A) (n = 4), and mean is shown. Bars ± SEM (**P<0.01 versus control). After subcutaneous matrigel injection in the presence and absence of PARP inhibitor DPQ, a decreased in VEGF-induced in vivo angiogenesis was observed. The formation of vessel in vivo was assessed after injection of HUVEC with matrigel plug contains VEGF and heparin. The neovascularizaton was evaluated by measurement of HB content of matrigel plug. The histogram represents the mean (n = 4) of the content, expressed as absorbance (DO)/100 mg of matrigel plug (B). (TIF) [file pgen.1003531.s001.tif]

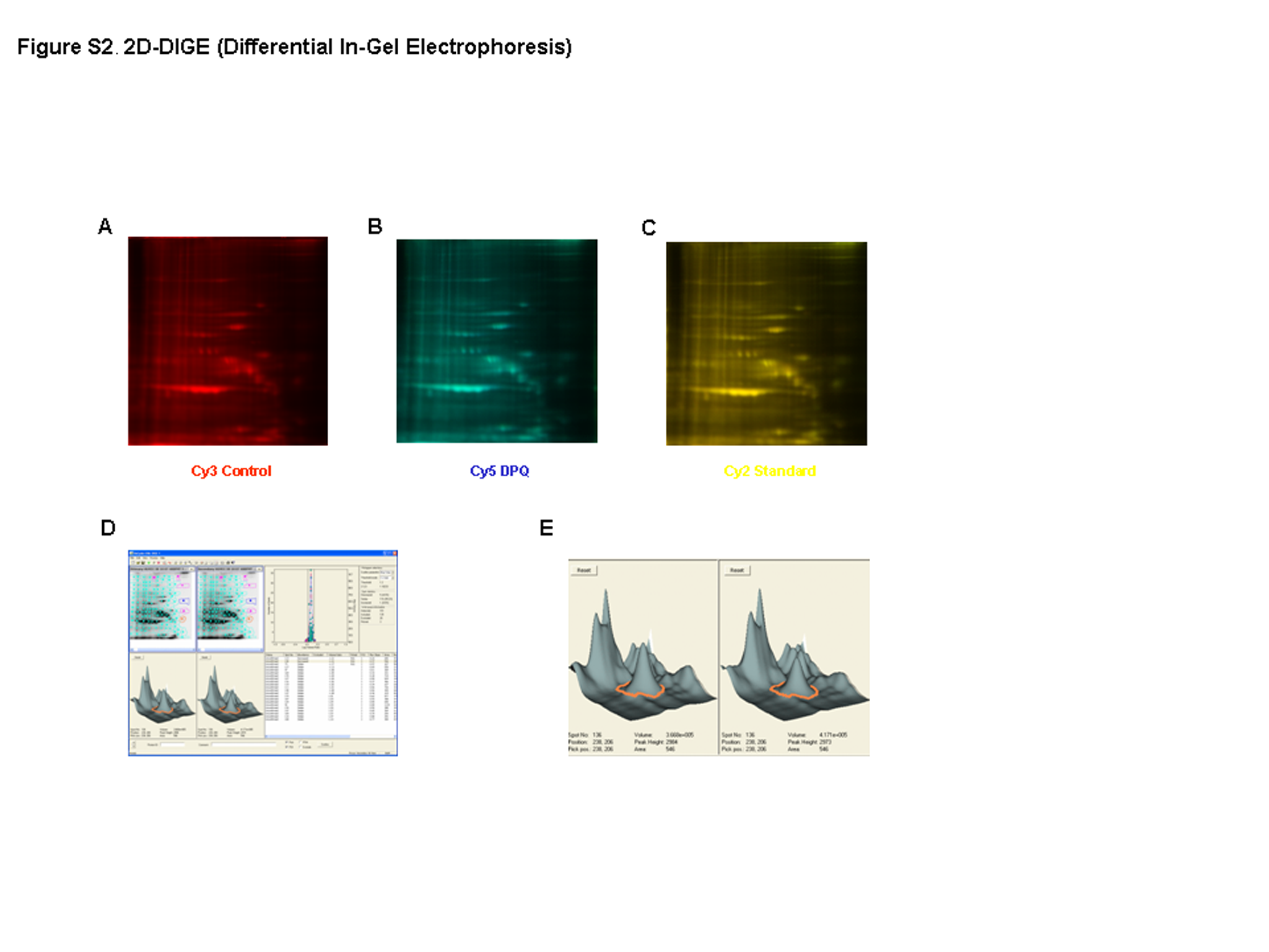

Supplement: Figure S2 — 2D-DIGE (Differential In-Gel Electrophoresis). HUVEC were solubilized in 2D-DIGE sample buffer (40 mM Tris, 7 M Urea, 2 M Thiourea, 1% ASB-14), sonicated and then the concentration was determined using the RC hDC Protein Assay (Bio- Rad). Fifty µg of protein was then labelled with 400 pmol of CyDye DIGE Fluor minimal dyes (GE Healthcare) and incubated on ice in the dark for at least 30 min according to manufacturer instructions Cy3 (A), Cy5 (B) for samples and Cy2 (C) for internal control consisting of equal parts of all samples). The reaction was halted by the addition of 10 mM lysine and incubated on ice for 10 min. Samples were loaded onto IPG strips (7 cm, pH 4–7) (Bio-Rad) by passive rehydration for 15 h and subjected to isoelectrofocusing using the PROTEAN IEF Cell System (Bio-Rad) according to the manufacturer's protocols. For the second dimension, strips were loaded on top of 7.5% polyacrylamide gels at 150 V for 1 h. The 2D gels were then scanned using a Typhoon Imager (GE Healthcare) at 100 µm resolution with λex/λem of 488/520, 532/580, and 633/670 nm for Cy2, Cy3, and Cy5, respectively. Image analysis was performed using DeCyder 6.5 software (GE Healthcare) as described in the user manual. Six independent experiments were performed for each experimental setup. Briefly, the differential in-gel analysis (DIA) module was used for spot detection, spot volume quantification and volume ratio normalization of different samples in the same gel (D). Differentially expressed spots were considered for identification based upon the fold change (>1.1) and the t-test (*P<0.05). (E) The Image analysis DeCyder Sofware indicated those differential spots detected in HUVEC treated with DPQ cells that were subsequently identified. (TIF) [file pgen.1003531.s002.tif]

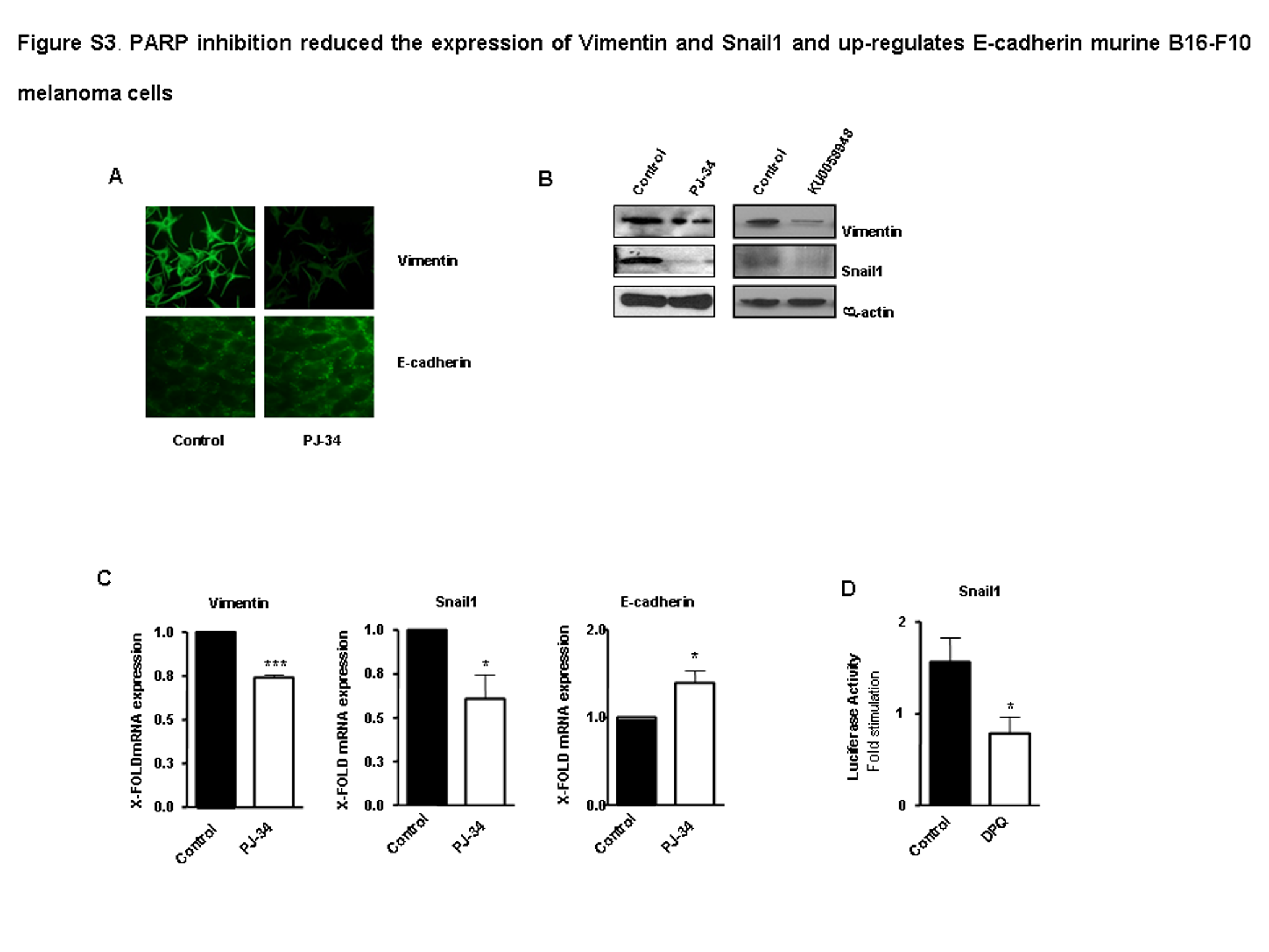

Supplement: Figure S3 — PARP inhibition reduced the expression of Vimentin and Snail1 and up-regulates E-cadherin murine melanoma cells. Cells were treated with either of the PARP inhibitors DPQ (40 µM) (not shown), PJ34 at 10 µM or KU0058948 (100 nM) during 22 hours. IF (A), western-blot (B) or qPCR (C) were performed to evaluate the impact of PARP inhibition on EMT markers. *P<0.05, ***P<0.001 PARP Inhibitor groups versus the control. β-actin was used as internal controls for protein loading. Luciferase activity (D) was determined after transfecting the constructions into the B16-F10 cells. *P<0.05 control versus DPQ. The expression of Firefly and Renilla luciferases was analyzed 48 h after transfection, according of the manufacturer's instructions. (TIF) [file pgen.1003531.s003.tif]

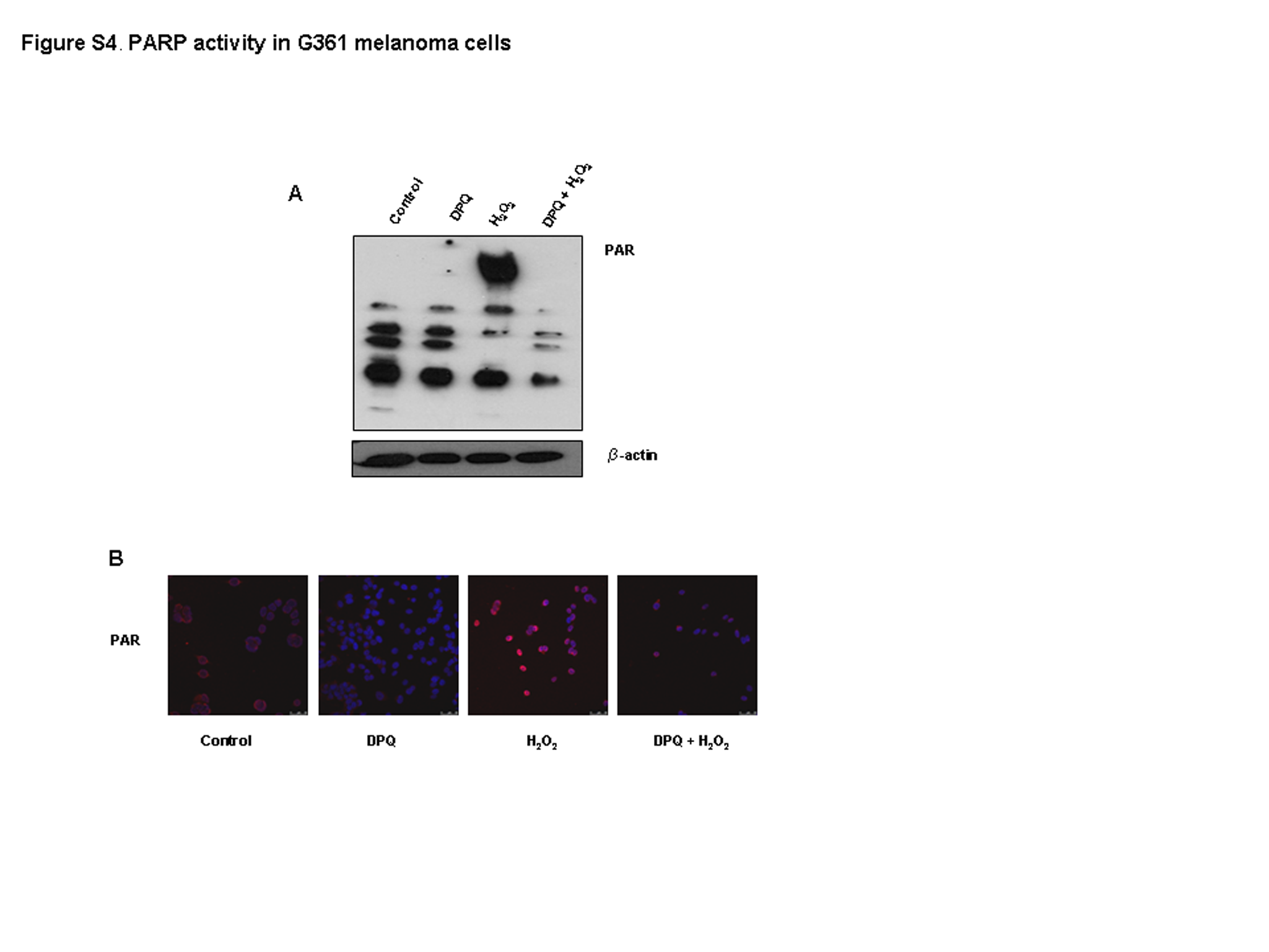

Supplement: Figure S4 — Western-blot (A) and immunofluorescence (B) of PARP activity inhibition in G361cells treated with the PARP-1 inhibitor, DPQ. (TIF) [file pgen.1003531.s004.tif]

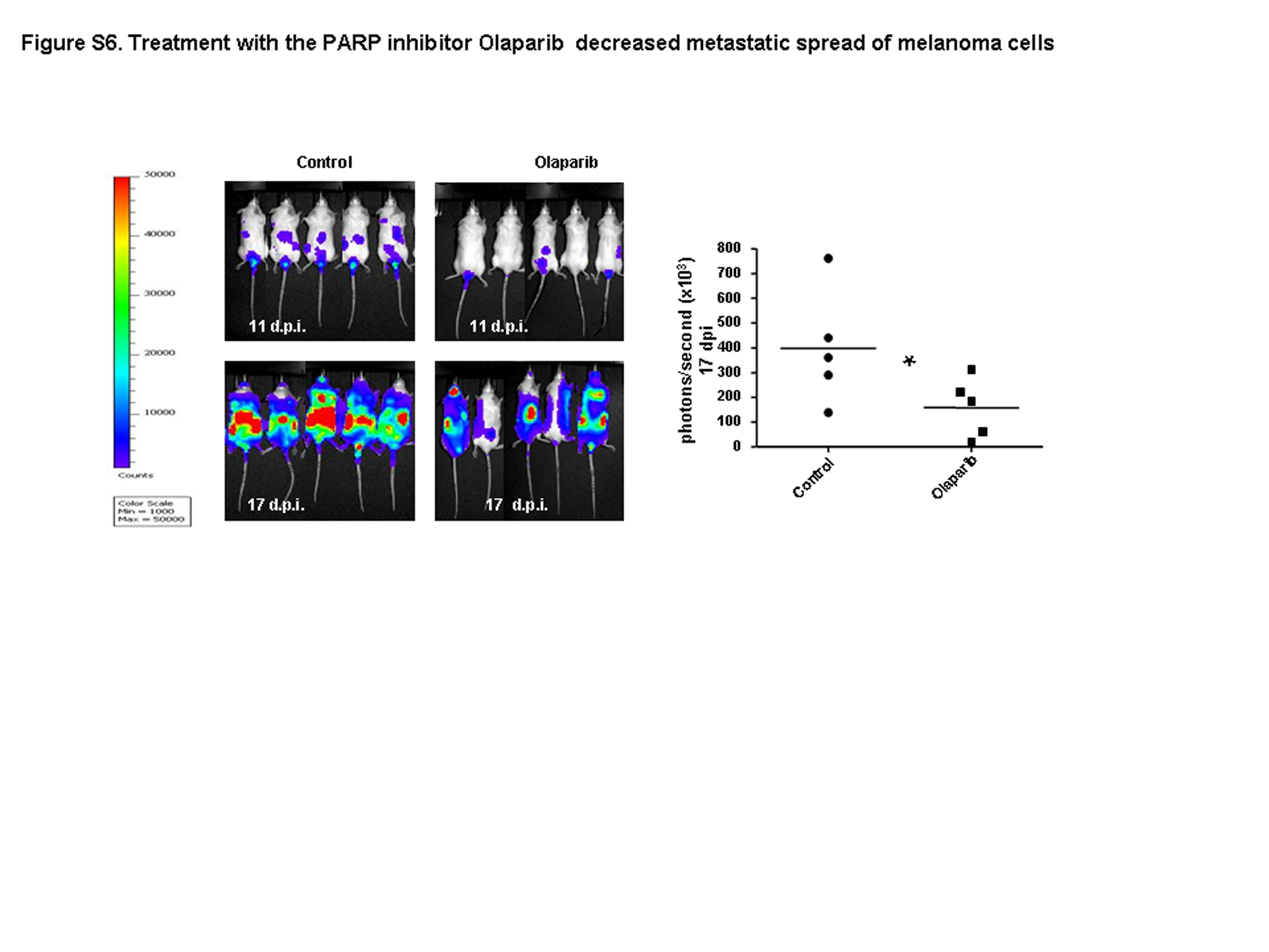

Supplement: Figure S6 — Treatment with the PARP inhibitor olaparib decreased metastatic spread of melanoma cells. C57BL/6 mice were inoculated with B16-F10-luc cells a treated with the PARP inhibitor olaparib (50 mg/kg) as explained in Methods. Results obtained on the 17th day are shown for quantitation. *P<0.05 olaparib versus control using the Mann-Whitney u-test. (TIF) [file pgen.1003531.s006.tif]

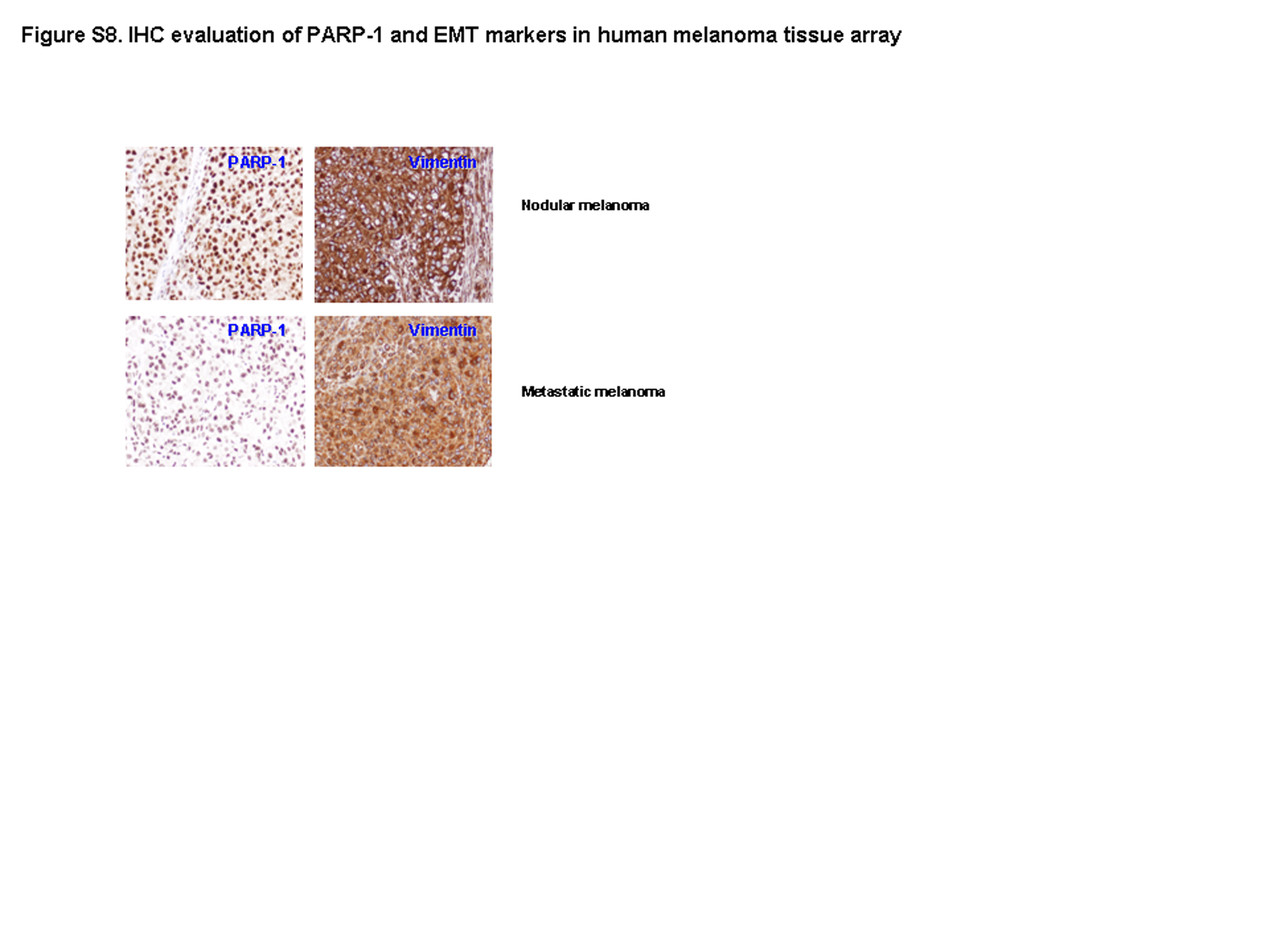

Supplement: Figure S8 — IHC evaluation of PARP-1 and EMT markers in human melanoma tissue array. Expression of PARP-1 and EMT markers in nodular and metastatic human melanoma. PARP-1 expression correlates with vimentin in nodular and metastatic melanoma. Snail1 and E-cadherin expression do not correlate with PARP-1 positivity. (TIF) [file pgen.1003531.s008.tif]
