# Supplementary material for: PARP-1 Regulates Metastatic Melanoma through Modulation of Vimentin-induced Malignant Transformation
Source: PLoS Genet. 2013 Jun 13;9(6):e1003531. doi: 10.1371/journal.pgen.1003531 (PMC3681683; doi:10.1371/journal.pgen.1003531)

Figure S5. Development of subcutaneous xenografts of melanoma is reduced by treatment with the PARP-1 inhibitor, DPQ

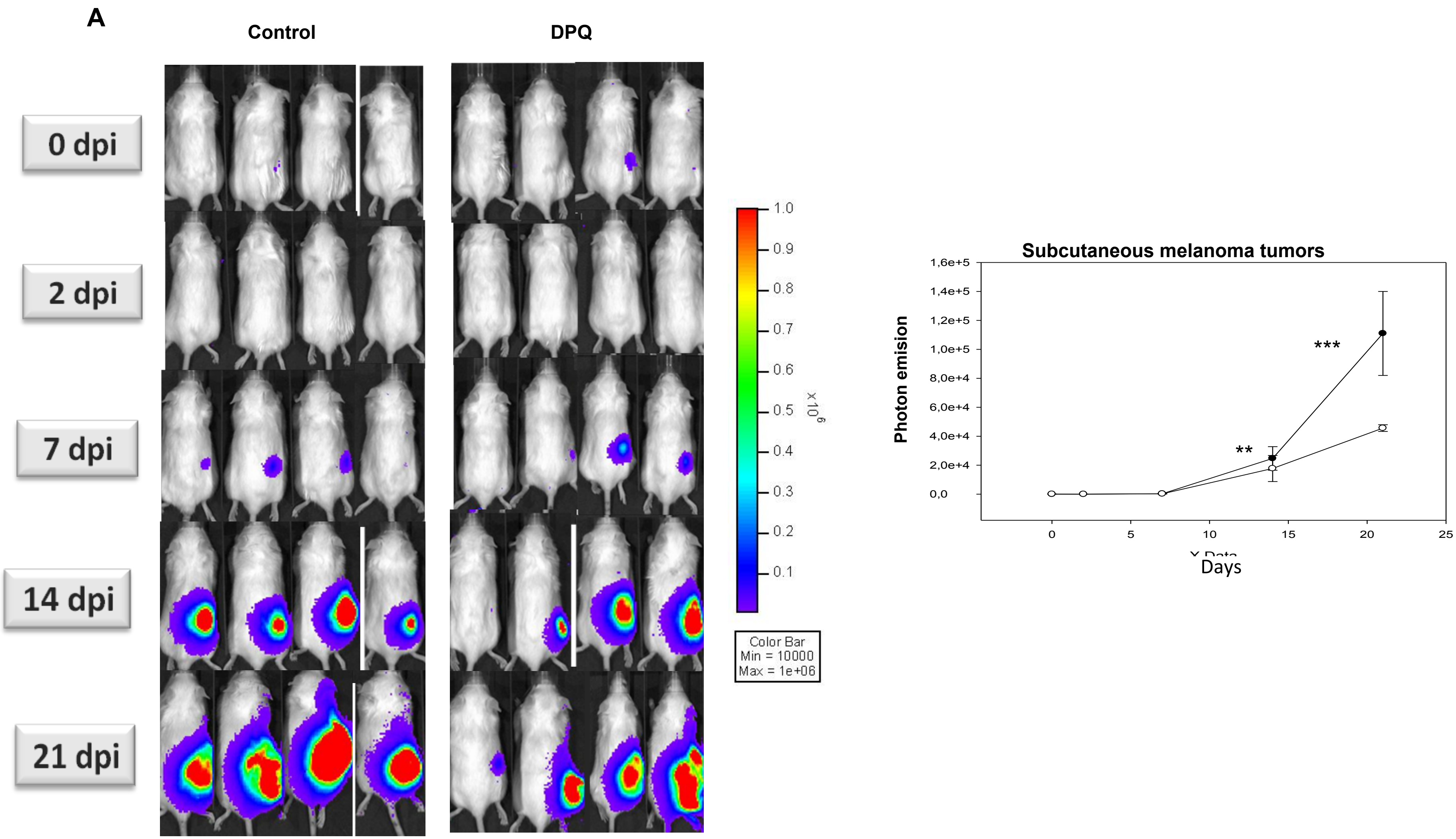

Tissue metastasis as function of PARP inhibition

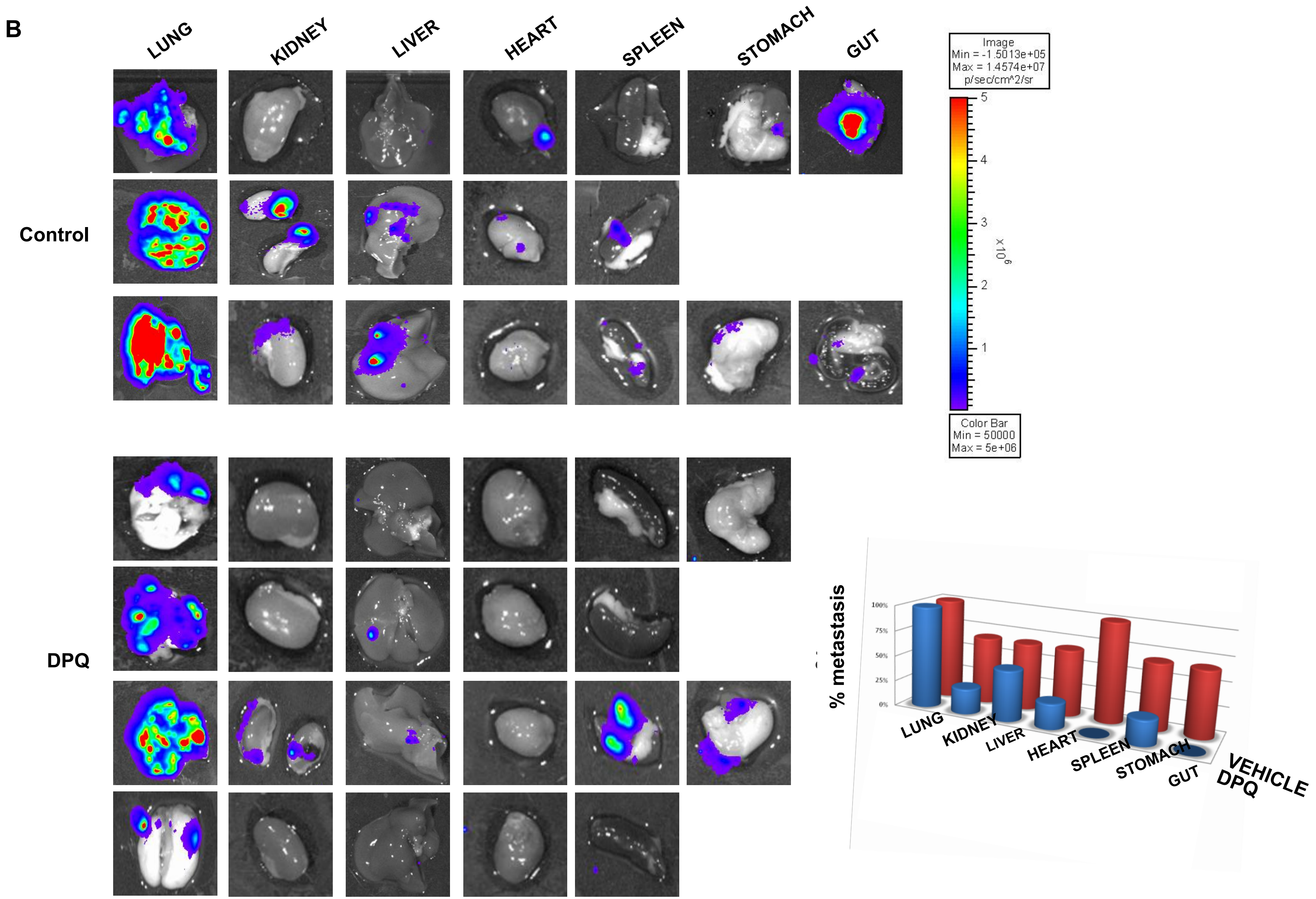

Supplement: Figure S5 — Development of subcutaneous xenografts of melanoma is reduced by treatment with the PARP inhibitor DPQ. (A) C57BL/6 albino mice (Jackson Laboratories, Philadelphia, USA), were inoculated with B16-F10-luc cells as explained in Methods. Localization and intensity of luciferase expression was monitored by in vivo bioluminescence imaging. Quantitation of luciferase activity over time in photons/s, is represented in the color bar. Vehicle (n = 4), DPQ (n = 4). **P<0.01; ***P<0.001. (B) Ex-vivo photon emission: treatment with the PARP inhibitor DPQ reduced lung and extra-pulmonary melanoma-induced metastasis. (PDF) [file pgen.1003531.s005.pdf]
