# Supplementary material for: PARP-1 Regulates Metastatic Melanoma through Modulation of Vimentin-induced Malignant Transformation
Source: PLoS Genet. 2013 Jun 13;9(6):e1003531. doi: 10.1371/journal.pgen.1003531 (PMC3681683; doi:10.1371/journal.pgen.1003531)
Supplement: Table S2 — Quantitation of western-blots. All western blots shown have been quantified using Quantity One software from Bio-Rad. (PDF) [file pgen.1003531.s010.pdf]

# Table S2: Western Blot quantitation

| Figure 1B | Vimentin | Snail1 | VE-cadherin |
|---|---|---|---|
| Control | 1 | 1 | 1 |
| DPQ | 0.25 | 0.26 | 1.4 |

| Figure 2B | Vimentin | Snail1 | E-cadherin |
|---|---|---|---|
| Control | 1 | 1 | 1 |
| PJ-34 | 0.88 | 0.42 | 1.15 |

| Figure 3A | PARP-1 | Vimentin | AXL | E-cadherin | Snail1 | ILK | β-catenin | pGSK-3β/GSK-3β |
|---|---|---|---|---|---|---|---|---|
| SCR | 1 | 1 | 1 | 1 | 1 | 1 | 1 | 1 |
| iPARP-1 | 0.31 | 1 | 1.16 | 1.11 | 0.11 | 0.02 | 0.67 | 0.76 |
| iVimentin | 0.11 | 0.12 | 0.09 | 1.23 | 0.95 | 0 | 0.81 | 0.39 |

| Figure 3B | PARP-1 | Vimentin | AXL | VE-cadherin | Snail1 | ILK | β-catenin | pGSK-3β/GSK-3β |
|---|---|---|---|---|---|---|---|---|
| SCR | 1 | 1 | 1 | 1 | 1 | 1 | 1 | 1 |
| iPARP-1 | 0 | 0.97 | 1.16 | 1.14 | 1.07 | 0.71 | 0.56 | 0.93 |
| iVimentin | 1.71 | 0.08 | 0 | 1.35 | 0.47 | 0.17 | 0.38 | 0.71 |

| Figure 4A | Vimentin | ILK | Snail1 | E-cadherin | β-catenin | pGSK-3β/GSK-3β |
|---|---|---|---|---|---|---|
| GFP | 1 | 1 | 1 | 1 | 1 | 1 |
| GFP-vimentin | 1.39 | 1.6 | 1.6 | 0.97 | 1.27 | 1.7 |

| Figure 4B | Vimentin | ILK | Snail1 | E-cadherin | β-catenin | pGSK-3β/GSK-3β |
|---|---|---|---|---|---|---|
| GFP | 1 | 1 | 1 | 1 | 1 | 1 |
| GFP + LiCl | 0 | 1.48 | 1.15 | 0.98 | 0.67 | 1.22 |
| GFP-vimentin | 1 | 1 | 1 | 1 | 1 | 1 |
| GFP-vimentin + LiCl | 2.65 | 0.99 | 1.28 | 0.9 | 2.21 | 1.07 |

| Figure 4C | Vimentin | ILK | Snail1 | E-cadherin | β-catenin | pGSK-3β/GSK-3β |
|---|---|---|---|---|---|---|
| GFP + SCR | 1 | 1 | 1 | 1 | 1 | 1 |
| GFP + iILK | 0 | 0.83 | 0 | 1.66 | 0.59 | 0.46 |
| GFP-vimentin + SCR | 1 | 1 | 1 | 1 | 1 | 1 |
| GFP-vimentin + iILK | 2.31 | 0.89 | 1 | 0.64 | 1.4 | 1.68 |

| Figure 5A | pVE-cadherin | VE-cadherin |
|---|---|---|
| Control | 1 | 1 |
| PJ-34 | 0.86 | 0.8 |
| KU0058948 | 0.8 | 0.25 |

| Figure S3B | Vimentin | Snail1 | E-cadherin |
|---|---|---|---|
| Control | 1 | 1 | 1 |
| PJ-34 | 0.9 | 0.48 | 1.2 |
| Control | 1 | 1 | |
| KU0058948 | 0.83 | 0.85 | |
